# Effects of changing population or density on urban carbon dioxide emissions

**DOI:** 10.1038/s41467-019-11184-y

**Published:** 2019-07-19

**Authors:** Haroldo V. Ribeiro, Diego Rybski, Jürgen P. Kropp

**Affiliations:** 1 Departamento de Física, Universidade Estadual de Maringá, Maringá, PR 87020-900 Brazil; 2 Potsdam Institute for Climate Impact Research – PIK, P.O. Box 601203, 14412 Potsdam, Germany; 3 Institute for Environmental Science and Geography, University of Potsdam, 14476 Potsdam, Germany

**Keywords:** Statistics, Statistical physics, thermodynamics and nonlinear dynamics, Climate sciences

## Abstract

The question of whether urbanization contributes to increasing carbon dioxide emissions has been mainly investigated via scaling relationships with population or population density. However, these approaches overlook the correlations between population and area, and ignore possible interactions between these quantities. Here, we propose a generalized framework that simultaneously considers the effects of population and area along with possible interactions between these urban metrics. Our results significantly improve the description of emissions and reveal the coupled role between population and density on emissions. These models show that variations in emissions associated with proportionate changes in population or density may not only depend on the magnitude of these changes but also on the initial values of these quantities. For US areas, the larger the city, the higher is the impact of changing its population or density on its emissions; but population changes always have a greater effect on emissions than population density.

## Abstract (web summary)

Approaches to examine the urbanization impact on climate change ignore that interactions between size and density may have an important influence on urban emissions. Here the authors show that variations in the emissions associated with changes in population or density may not only depend on the magnitude of these changes but also on the initial values of these quantities.

## Introduction

Carbon dioxide (CO_2_) emissions are considered one of the main causes of Earth’s climate change^1^. Despite covering only 0.4–0.9% of global land surfaces^2^, urban areas are responsible for more than 70% of such emissions^3,4^. This fact assigns cities a central role in pursuing solutions and mitigation strategies for the global climate change problem. Because of that, researchers from several disciplines have investigated the effects of urbanization on CO_2_ emissions^5–20^. The question of whether urbanization promotes or mitigates climate change is ubiquitous among these works, and the approaches to probe such issues differ, but can be roughly organized into two groups.

Underlying the first approach, there is the so-called urban scaling hypothesis^21,22^, which states that city emissions (*C*) are described by a power-law function of population size (*P*), that is, *C* ~ *P*^*β*^, where *β* is the urban scaling exponent or the scale-invariant elasticity. For CO_2_ emissions in the United States (US), researchers have reported a 1:1 relationship (*β* ≈ 1, constant returns to scale) with the population of metropolitan areas^8^, while the same quantity was found to scale superlinearly (*β* = 1.46, increasing returns to scale) when defining the US cities as connected urban spaces^9^. When considering local air pollution, an exponent *β* ≈ 3/4 (decreasing returns to scale) was observed for US metropolitan areas^19^. There is also evidence supporting the idea that the scaling between emissions and population depends on the degree of economic development of the urban systems, with increasing returns to scale (*β* > 1) observed for cities of developing countries and economy of scale (*β* < 1) for developed ones^17^. The second approach is focused on the understanding how population density affects CO_2_ emissions per capita^5,6,12,14,15,23^, that is, to investigate the relationship between *C*/*P* and *P*/*A*, where *A* stands for the urban unit area. A recent work has proposed that CO_2_ emissions per capita (related to buildings and on-road sectors) and population density are related via the power-law *C*/*P* ~ (*P*/*A*)^*α*^, with an exponent *α* ≈ −0.8 for the US urban areas^15^.

Although these two bodies of the urban CO_2_ literature are strongly linked by the purpose of understanding how urbanization affects climate change, they have operated widely independent from each other, and their approaches are perceived as different issues. Researchers using urban scaling are assuming population size as the most relevant urban feature for describing CO_2_ emissions, while those working with the per capita density scaling consider population density as the most significant covariate. Both approaches, however, have produced controversial results regarding the influence of population or population density on urban emissions (see, for instance, refs. ^7,12^ and Supplementary Table 1). Large part of these discrepancies can be attributed to different methodologies for estimating CO_2_ emissions and defining the boundaries of urban areas, but also because both approaches ignore that population and area are correlated^24,25^ and the influence of a possible interconnected role between these quantities on urban emissions.

Inspired by the economic theory of production functions^26^, we propose here a new approach for investigating emissions in urban areas that simultaneously considers the effects of population and area along with possible interactions between these urban metrics. We show that our models recover the two conventional approaches when ignoring the effects of urban area (urban scaling) or when assuming that the emissions display constant returns to scale with population and area (per capita density scaling). When compared with the two conventional approaches, our models provide a significantly better description for the emissions in US urban areas. These results confirm the predictive power of the interactions between population and area, which in turn have intriguing consequences about the effect of these quantities on urban emissions. Our approach indicates that emissions may display decreasing or increasing returns to scale with population and area depending on whether the product *P* × *A* exceeds a particular threshold. We further find that the impact of a proportionate change in the population and density of a city on its emissions increase with its area but always have decreasing returns to scale; moreover, changes in population always have more impact on emissions than changes in density.

## Results

### Urban scaling and per capita density scaling of emissions

We start by revisiting how population scaling and per capita density scaling approaches have been applied for investigating CO_2_ emissions in urban areas. To do so, we used the same dataset reported by Gudipudi et al.^15^ which comprises CO_2_ emissions (sum of on-road and building emissions) in US urban areas in the year 2000. As described in Methods section, this dataset is constructed by combining gridded data from different sources, and by applying the city clustering algorithm^27^ for defining the urban units. There are a total of 3285 urban units and for each one we have population (*P* in raw counts), area (*A* in km^2^), and CO_2_ emissions (*C* in tonnes of CO_2_).

**Fig. 1:**
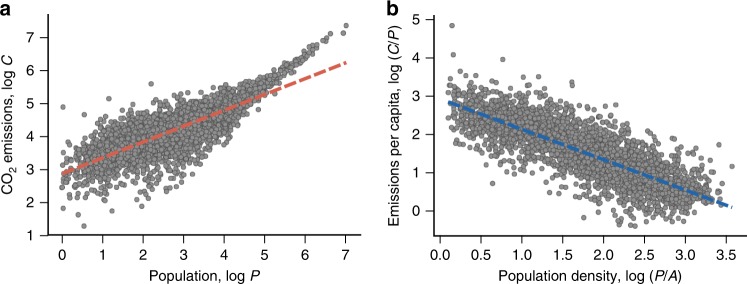
Conventional approaches for investigating urban emissions. **a** Urban scaling: the scaling relationship between CO_2_ emissions (*C*) and population size (*P*). The dashed line represents a power-law fit (Eq. (1)) with an exponent *β* = 0.48 ± 0.01. We observe that this model underestimates the emissions for large population sizes. **b** Per capita density scaling: the scaling law between CO_2_ emissions per capita (*C*/*P*) and population density (*P*/*A*). The dashed line is a power-law fit (Eq. (2)) with an exponent *α* = −0.79 ± 0.01. In both plots, each dot is associated with a US urban unit obtained from the city clustering algorithm (see Methods) and all quantities are expressed in base-10 logarithmic scale. Emissions are measured in tonnes of CO_2_, population in raw counts, and area in square kilometers

Having defined our variables, within the urban scaling framework, CO_2_ emissions and population size are related via the power-law function

1 $$C\sim P^\beta ,$$

where *β* is the scaling exponent. To estimate the parameter *β*, we have applied the usual least-squares method to the relationship between log*C* and log*P*. This approach leads to *β* = 0.48 ± 0.01 (*p*-value = 0, permutation test, Supplementary Fig. 1) and the relationship between both variables (on logarithm scale) is shown in Fig. 1a. At a cursory glance, this value of *β* indicates a sublinear trend between emissions and population, so that a 1% increase in the population level of a city associates with only 0.48% increase in its CO_2_ emissions. However, a closer inspection of Fig. 1a shows that Eq. (1) deviates systemically from the data and underestimates the emissions in large populated areas. In addition to that, the exponent *β* is likely to be biased by the confounding effect of area because population and area of urban units are correlated to each other via a power-law relation^24,25^.

On the other hand, within the per capita density scaling framework, the relationship between CO_2_ emissions per capita and population density is described by the power-law function^15^

2 $$C/P\sim (P/A)^\alpha ,$$

where *α* is another scaling exponent. Figure 1b illustrates this relationship on logarithmic scale (log*C*/*P* versus log*P*/*A*) from which we have estimated *α* = −0.79 ± 0.01 (*p*-value = 0, permutation test, Supplementary Fig. 1) via ordinary-least-squares method. We observe that Eq. (2) is slightly better than Eq. (1) for describing our data (Supplementary Fig. 2), and does not seem to exhibit any systematic deviation. Our estimate for *α* is in agreement with the results reported by Gudipudi et al.^15^, and indicates that every 1% increase in the population density of a city associates with a 0.79% reduction in its CO_2_ emissions per capita. However, and similarly to the urban scaling case, the exponent *α* is also likely to be biased by the confounding effect of population, since density and population values are also correlated^24,25^.

In order to improve the description of urban emissions, we propose here an analogy with the economic theory of production functions. This theory has a central role in several branches of economics^26^, and in general terms, a production function establishes a mathematical relationship between inputs (capital, labor, land, etc.) and output of goods (iron, cars, wheat, etc.) in some production process. Using this mathematical description, economists may ask how much output can be produced with particular combinations of inputs, and what are the alternatives (in terms of inputs) for producing a particular good. These ideas transpose well into our context assuming CO_2_ emissions as the output and population and area as the inputs in a production process mediated by cities. Similarly to a two-factor production model, we thus consider that *C* = *F*(*P*, *A*), where *F*(…) stands for the form of the production function. By putting this analogy forward, we establish a more general approach for modeling urban emissions that simultaneously accounts for the effects of population and area along with possible interactions between these urban metrics. As we shall verify, we can borrow not only functional forms from the theory of production functions but also key concepts that are very useful in the context of urban emissions (see Methods).

### Cobb–Douglas model of urban carbon emissions

We start this analogy with the Cobb–Douglas model^28^, arguably the most widely known and used production function^26^. In our case, it takes the form

3 $$C\sim P^{\beta _P}\,A^{\beta _A}\,{\mathrm{or}}\,\,{\mathrm{log}}\,C\sim \beta _P{\mathrm{log}}\,P + \beta _A{\mathrm{log}}\,A,$$

where *β*_*P*_ and *β*_*A*_ are two independent exponents. We immediately notice that this model recovers the urban scaling (Eq. (1)) if *β*_*P*_ = *β* and *β*_*A*_ = 0 (that is, when ignoring the effect of area) and the per capita density scaling (Eq. (2)) if *β*_*P*_ = *α* + 1 and *β*_*A*_ = −*α*. We further remark that the Cobb–Douglas model can be obtained from Eqs. (1) and (2) if we consider the empirical relation between population and area^24,25^ (see Methods). Similarly to the models of Eqs. (1) and (2), the Cobb–Douglas function exhibits a scale-invariant elasticity *ε* = *β*_*P*_ + *β*_*A*_ (see Methods), meaning that a proportionate increase in emissions associated with a proportionate increase in population and area is independent of *P* and *A*. Thus, when *β*_*P*_ + *β*_*A*_ < 1 there are decreasing returns to scale (doubling *P* and *A* implies less than doubling *C*), whereas if *β*_*P*_ + *β*_*A*_ > 1 there are increasing returns to scale (doubling *P* and *A* implies more than doubling *C*), and only for *β*_*P*_ + *β*_*A*_ = 1 this model presents constant returns to scale (doubling *P* and *A* implies exactly doubling *C*). Thus, we notice that Eq. (2) is a particular case of the Cobb–Douglas model with constant returns to scale. On the other hand, without any constraint for the exponents *β*_*P*_ and *β*_*A*_, the Cobb–Douglas model represents a genuine generalization that cannot be related to Eqs. (1) and (2). In addition to that, we can interpret Eq. (3) as the result of accounting for the confounding effect of area *A* within the urban scaling framework (Eq. (1)) via a multiple linear regression (in log-transformed variables).

**Fig. 2:**
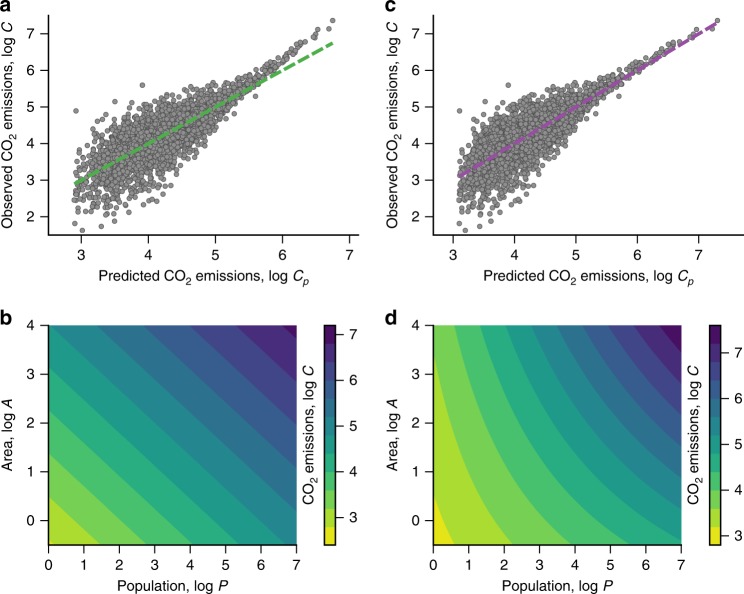
The interplay between population and area on CO_2_ emissions. **a** Scatter plot of the observed values of CO_2_ emissions (*C*) and those predicted (*C*_*P*_) by the Cobb–Douglas model (Eq. (3) with *β*_*P*_ = 0.31 ± 0.01 and *β*_*A*_  = 0.45 ± 0.03). This model is a significantly better fit when compared with the urban scaling and the per capita density scaling models (Supplementary Fig. 2). **b** A contour plot of Eq. (3) as a function of *P* and *A* on logarithmic scale. The straight isolines/isoquants show how population and area must change in order to keep the emissions unchanged. **c** Scatter plot between the observed and predicted CO_2_ emissions obtained from the translog model (Eq. (5) with *β*_*P*_ = 0.28 ± 0.02, *β*_*A*_ = 0.14 ± 0.05, and *β*_*C*_ = 0.07 ± 0.01). This model further refines the goodness of the predictions (Supplementary Fig. 2), particularly reducing the bias in urban areas with high emissions. **d** A contour plot of Eq. (5) as a function of *P* and *A*. We note that the isolines/isoquants of this model are not straight lines as those from the Cobb–Douglas model. We have employed base-10 logarithmic quantities in all panels

Although the model of Eq. (3) may represent a better description for CO_2_ emissions, it introduces some drawbacks related to the use of ordinary-least-squares for finding the best fitting parameters *β*_*P*_ and *β*_*A*_. This happens because population and area are correlated to each other, a problem known as multicollinearity and that can lead to unstable estimates for the model parameters. As detailed in Methods, we have applied the regularization approach of the ridge regression^29,30^ in order to account for this problem. To state briefly, the ridge regression adds a penalty/regularization term proportional to the square of the magnitude of coefficients upon the residual sum of squares, which in turn stabilizes the regression coefficients and accounts for the multicollinearity. This approach yields *β*_*P*_ = 0.31 ± 0.01 and *β*_*A*_ = 0.45 ± 0.03 (*p*-values = 0, permutation test, Supplementary Fig. 3), and Fig. 2a shows the relationship between the actual values of the CO_2_ emissions and those predicted by Eq. (3). We have verified that the Cobb–Douglas model provides a significantly better fit to our data (Supplementary Fig. 2) when compared with the urban scaling (Eq. (1)) and the per capita density scaling approaches (Eq. (2)). Moreover, the fact that *β*_*P*_ is much smaller than *β* reinforces the idea that the urban scaling approach is indeed affected by the confounding effect of the area.

Because *β*_*P*_ + *β*_*A*_ < 1, our results indicate that CO_2_ emissions display diminishing returns as population and area are incrementally increased by the same factor (that is, keeping density constant). In particular, our estimates indicate that every 1% increase in both the population and area of a city associates with a 0.76% increase in its emissions. The interconnected role of population and area on CO_2_ emissions is better visualized in Fig. 2b, where we depict a contour plot of Eq. (3) on logarithmic scale. In this representation, the isoquants (or isolines) are described by straight lines [log*A* ~ −(*β*_*P*_/*β*_*A*_) log*P*] and show how population and area must change to keep emissions constant. These isoquants also indicate that if the population of a city increases, its population density must also increase to keep emissions unaltered. This behavior is better understood by rewriting Eq. (3) as $C\sim P^{\beta _P + \beta _A}(P/A)^{ - \beta _A}$ and noticing that *β*_*P*_ > 0 and *β*_*A*_ > 0 (Supplementary Fig. 4 shows the contour plot in terms of population density). From this form of Eq. (3), we also conclude that a proportionate change in population has more impact on CO_2_ emissions than a proportionate change in density since |*β*_*P*_ + *β*_*A*_| > |*β*_*A*_|. For a particular urban unit in our data, this means that if its population decreases by 1% while its density remains constant (that is, a 1.01% increase in its area), the model predicts a 0.76% reduction in its CO_2_ emissions; whereas a 1% raise in its density while population is unaltered (that is, a 1% reduction in its area), implies only 0.45% decrease in its CO_2_ emissions.

Another interesting aspect of an isoquant is its slope, a quantity known as the technical rate of substitution in economics^26^ (see Methods). In our context, this slope measures how much the population of a city should change in response to alterations in its area in order to keep the same level of emissions. For Eq. (3), the slopes of the isoquants are $\frac{{dA}}{{dP}} = - \frac{{\beta _P}}{{\beta _A(P/A)}}$, and thus, they are completely determined by the city density (assuming that *β*_*P*_ and *β*_*A*_ are known). If we consider a logarithmic scale, these slopes are equal to $\frac{{d\,{\mathrm{log}}\,A}}{{d\,{\mathrm{log}}\,P}} = - \beta _P/\beta _A$ regardless of the values of *P* and *A* (as we see in Fig. 2b). In economics, these isoquants are also analyzed in terms of the so-called elasticity of substitution^26^ (see Methods), a dimensionless measure that (mapped to our case) quantifies the efficiency at which population and area substitute each other, and that somehow reflects the shape of the isoquants^26^. Usually, more L-shaped isoquants are associated with low elasticity of substitution (that is, there is no room for replacing *A* by *P* while keeping emissions constant), whereas more linear/smooth isoquants tend to have high elasticity of substitution (it is easy to replace *A* by *P* while keeping emissions constant). The Cobb–Douglas model has unitary elasticity of substitution, regardless of the values of *P*, *A*, and *C*, and also the exponents *β*_*P*_ and *β*_*A*_^26^. Thus, although the Cobb–Douglas model provides a better fit to our data (compared with Eqs. (1) and (2)), it also makes a series of assumptions that do not have any compelling reasons to hold true in urban systems (as it also happens in economics^26^). Moreover, Fig. 2a shows that Eq. (3) has a bias for large values of *C*, which indicates that relaxing some underlying assumptions of the Cobb–Douglas model may lead to a better description of the emissions.

We first relax the condition of unitary elasticity of substitution by considering a model based on the constant elasticity of substitution (CES) production function^26,31^

4 $$C\sim (\beta _PP^{ - \gamma } + \beta _AA^{ - \gamma })^{ - 1/\gamma },$$

where *γ* is a parameter and *β*_*P*_ + *β*_*A*_ = 1. The CES model emerged as a generalization of the Cobb–Douglas function (which is recovered when *γ* → 0) exactly because economists have considered the assumption of unitary elasticity of substitution as unduly restrictive^31^. As the name suggests, the CES model has a constant elasticity of substitution equal to 1/(*γ* + 1), and thus, by varying *γ* we have a wide range of possible elasticities. We have adjusted Eq. (4) to our data using the Levenberg–Marquardt algorithm. However, these fits are very problematic because of the non-linear nature of the model. Depending on the initial guess used for the parameters, we find very unstable and meaningless results. These fits also lead to very large variations in the parameters when applying a resampling strategy to our data. Furthermore, we have observed that even when the fits converge, the CES model does not represent the best description to our data. In addition to the non-linearity, this happens because the CES model also carries crucial assumptions that appear not to hold in our case. This model has unitary elasticity of scale (*ε* = 1, that is, it assumes that by doubling *P* and *A* implies doubling *C*), and similarly to Cobb–Douglas, the slopes of the isoquants $\left( {\frac{{dA}}{{dP}} = - \frac{{\beta _P}}{{\beta _A(P/A)^{\gamma + 1}}}} \right)$ are determined solely by the city density.

### Transcendental logarithm model of emissions

To overcome these limitations and constraints, we considered a more general production function known as the transcendental logarithm (translog) model^26,32^. Just like the CES is a natural extension of the Cobb–Douglas function, the translog model represents the next logical step towards a more flexible function for modeling the CO_2_ emissions in terms of *P* and *A*. This model is written as

5 $${\mathrm{log}}\,C\sim \beta _P\,{\mathrm{log}}\,P + \beta _A\,{\mathrm{log}}\,A + \beta _C\,{\mathrm{log}}\,(P)\,{\mathrm{log}}\,(A),$$

where *β*_*C*_ is an additional parameter. Different from Eqs. (3) and (4), the translog model has a non-constant elasticity of scale [*ε* = *β*_*P*_ + *β*_*A*_ + *β*_*C*_ log (*PA*)], meaning that proportionate changes in the emissions associated with proportionate changes in population and area depend on the initial values of *P* and *A*. Furthermore, the elasticity of substitution of Eq. (5) varies with *P*, *A*, and *C*, and the isoquant slope depends on *P* and *A* (see Methods). By comparing the translog model with the Cobb–Douglas, we notice that all this additional flexibility is solely related to the inclusion of the interaction term [*β*_*C*_ log (*P*) log (*A*)] between population and area, where *β*_*C*_ quantifies the intensity of this interaction. It is this interaction term that allows the effect of population and area on emissions to vary with *P* and *A*. This effect is better understood in terms of the marginal products^26^ (see Methods), an economic quantity that mapped to our context represents the response in CO_2_ emissions caused by changes in population or area. The marginal product of population (in logarithmic scale) is defined as $\frac{{d\,{\mathrm{log}}\,C}}{{d\,{\mathrm{log}}\,P}} = \beta _P + \beta _C\,{\mathrm{log}}\,A$, while the marginal product of area is $\frac{{d\,{\mathrm{log}}\,C}}{{d\,{\mathrm{log}}\,A}} = \beta _A + \beta _C\,{\mathrm{log}}\,P$. Thus, for instance, we observe that the marginal product of population depends on the area of the urban unit. This behavior contrasts with the Cobb–Douglas predictions (corresponding to *β*_*C*_ = 0), in which the marginal products are independent of *P* and *A*.

As in the Cobb–Douglas case, we have considered the ridge regression approach in order to account for the multicollinearity and adjust Eq. (5) to our data (see Methods). This approach yields *β*_*P*_ = 0.28 ± 0.02, *β*_*A*_ = 0.14 ± 0.05, and *β*_*C*_ = 0.07 ± 0.01 (*p*-values = 0, permutation test, Supplementary Fig. 3). We further note that the translog model is a significantly better fit to our data when compared with the previously discussed models (Supplementary Fig. 2). This fact is highlighted in Fig. 2c, where we observe that the model of Eq. (5) refines the quality of the predictions and reduces the bias observed for Eq. (3) in urban areas with high emissions. We further remark that all fitted parameters of Eq. (5) are significantly different from zero, confirming the predictive power of the interaction term between population and area. Therefore, in addition to improving the description of the CO_2_ emissions, Eq. (5) reveals that the effect of population and area on the emissions intensifies with the increase of urban population and area. This becomes clear by noticing that the elasticity of scale increases with *P* and *A* [*ε* = 0.42 + 0.07 log (*PA*)], that is, the more populous and the more widespread a city is, the larger is the impact of a proportionate change in its population and area (a growth with constant density) on its emissions. It is further intriguing to notice that, because the elasticity of scale varies with *P* and *A*, the translog model displays decreasing, increasing, or constant returns to scale depending on whether the product Ω = *PA* is, respectively, smaller, larger, or equal to the critical value

6 $${\mathrm{\Omega }}^ \ast = 10^{\frac{{1 - \beta _P - \beta _A}}{{\beta _C}}}.$$

For the US data Ω* ≈ 1.93 × 10^8^, and thus, cities having Ω < Ω* display decreasing returns to scale, while those having Ω > Ω* feature increasing returns to scale. For instance, the translog model predicts that a 1% increase in population and area of a large city with *P* = 8 × 10^6^ and *A* = 6000 km^2^ (roughly the size of Chicago, Ω = 4.8 × 10^10^) associates with 1.17% raise in its emissions, whereas the same change in a relatively small city with *P* = 90,000 and *A* = 140 km^2^ (roughly the size of Santa Fe, Ω = 12.6 × 10^6^) associates with only 0.92% raise in its emissions.

The interconnected role of population and area on CO_2_ emissions is better visualized in the contour plot of Eq. (5) shown in Fig. 2d. In comparison with Fig. 2b, we note that the interaction term bends the isoquants upward and make their slopes $\left[ {\frac{{dA}}{{dP}} = \frac{{ - 1}}{{(P/A)}}\left( {\frac{{\beta _P + \beta _C\,{\mathrm{log}}\,A}}{{\beta _A + \beta _C\,{\mathrm{log}}\,P}}} \right)} \right]$ a function of *P* and *A*, and not only of the city density as in the Cobb–Douglas model. We further observe that the spacing between the isoquants representing equally incremented values of log*C* changes with the values of log *P* and log *A*. This behavior contrasts with the equally spaced isoquants produced by the model of Eq. (3), and emphasizes that proportionate changes in emissions caused by changes in both population and area depend not only on the intensity of the changes but also on the initial values of population and area.

In terms of density, we can rewrite Eq. (5) as

7 $$C\sim P^{\beta _P + \beta _A + \beta _C\,{\mathrm{log}}\,P}(P/A)^{ - \beta _A - \beta _C\,{\mathrm{log}}\,P},$$

and from this expression, we find that the elasticity of scale in terms of population and density is *ε* = *β*_*P*_ + *β*_*C*_ log *A*. Thus, the more widespread a city is, the larger is the impact of a proportionate change of population and density (that is, area remains constant) on its emissions. In a concrete example, our estimates indicate that a 1% raise in population and density of a large city with 6000 km^2^ associates with a 0.54% increase in its emissions, while the same change in a city with 140 km^2^ correlates with a 0.42% raise in its emissions. Furthermore, in terms of population and density, increasing returns to scale (*ε* > 1) is only possible for urban areas exceeding the critical value $A^ \ast = 10^{(1 - \beta _P)/\beta _C}$. For the US data, A* ≈ 1.93× 10^10^ km^2^, an area roughly corresponding to 38 times the area of Earth. Therefore, our estimates indicate that only decreasing returns to scale are possible when the CO_2_ emissions are described in terms of population and density.

The translog model of Eq. (7) also allows us to verify whether changes in population have more impact on the emissions than changes in density. To do so, we have compared the absolute values of the marginal products of population $\left[ {\frac{{d\,{\mathrm{log}}\,C}}{{d\,{\mathrm{log}}\,P}} = \beta _P + \beta _A + \beta _C\,{\mathrm{log}}(PA)} \right]$ and density $\left[ {\frac{{d\,{\mathrm{log}}\,C}}{{d\,{\mathrm{log}}\,(P/A)}} = - \beta _A - \beta _C\,{\mathrm{log}}\,P} \right]$. The marginal product of population represents the response in emissions associated with changes in population when density remains constant, whereas the marginal product of density expresses the response in emissions caused by a change in density when population remains constant. The inequality $\left| {\frac{{d\,{\mathrm{log}}\,C}}{{d\,{\mathrm{log}}\,P}}} \right| > \left| {\frac{{d\,{\mathrm{log}}\,C}}{{d\,{\mathrm{log}}\,(P/A)}}} \right|$ simplifies to $A > A^ \ast = 10^{ - \beta _P/\beta _C}$ when *β*_*P*_, *β*_*A*_, *β*_*C*_ > 0 and *A*, *P* > 1 (assumptions that agree with our estimates). By plugging the estimated values of *β*_*P*_ and *β*_*C*_, this condition becomes *A* > 10^−4^ km^2^, and hence we conclude that population size always have more impact on emissions than changes in population density. We have reached the same conclusion with the simpler model of Eq. (3). However, and as we have verified, the translog approach further refines the description of CO_2_ emissions and indicates that the impact of population and density on the emissions changes with the population and density of the cities. For instance, according to our estimates for the US data, a 1% increase in the density of a town with 10,000 inhabitants associates with a 0.42% reduction in its emissions, while the same change in a larger city with 1 million inhabitants associates with a 0.56% reduction in its emissions. This behavior contrasts with results of Eq. (3), whose predictions related to changes in the density of a city are independent of its population. By carrying this predictions forward and in line with other studies^33^, our results suggest that the densification of large populated urban areas is likely to have important contributions to the reduction of urban CO_2_ emissions.

## Discussion

We have shown that two conventional approaches used to study the effect of urbanization on urban CO_2_ emissions suffer from confounding effects, and are unable to describe the interconnected role of population and area on urban emissions. Inspired by the economic theory of production functions, we have proposed new models for describing urban emissions simultaneously in terms of the population and area (or population density) of urban units. These models not only account for such confounding factors but significantly refine the description of the emissions in terms of urban quantities. In addition to being better fits to data, our models reveal intriguing aspects about the interplay between population and area (or density) on urban emissions that would be entirely neglected under the urban scaling or the per capita density scaling frameworks. Among these findings, our results indicate that variations in emissions associated with proportionate changes in population and area do not only depend on the magnitude of these changes but also on the product Ω = *PA*. In particular, depending on whether Ω exceeds or not the critical value Ω*, urban emissions can display increasing (Ω > Ω*) or decreasing returns to scale (Ω < Ω*) with population and area. When described in terms of population and density, we have found that urban emissions display decreasing returns to scale, meaning that doubling population and density of a city always associates with less than doubling its emissions. We have further verified that changing the population of a city has more impact on its emissions than changing its density. In spite of that and in general terms, our models define conditions under which changes in population have more impact than changes in density (or vice versa) on emissions and further predict a transition-like behavior where the dominant role changes between these quantities if the urban area exceeds a threshold value (A*).

Our work has, however, its limitations in a sense that ideally the comparison between the effects of population and area (or density) on the emissions should be made after accounting for every other factor (such as economic activity, technology, and even individual attitudes) that possibly affects urban emissions. Thus, while our models account for the confounding effects of area (and density), the emissions may also be affected by other confounding factors not available in our dataset. One possibility for addressing this problem would be to include further control variables in our models, an approach that somehow resembles the IPAT equations^34–36^, a framework proposed to model environmental impact (I) as the product of population (P), affluence (A), and technology (T), but with the advantage of considering population density (or area) as a predictor and allowing the interactions among such factors. Another possibility for overcoming these possible confounding effects is to combine our approach with the recently proposed urban Kaya scaling^20^ that relates CO_2_ emissions, population, gross domestic product, and energy consumption. Combining these different approaches into a single and coherent framework could represent an exciting perspective for solving the economics of urban CO_2_ emissions and defining its most important covariates. However, such endeavors require homogeneous and consistent data, which are still scarce on large spatial scales. While moving from urban units defined in terms of connected urban spaces to some political or administrative divisions would be a possibility, this approach is likely to introduce serious bias to the empirical estimates^37^ in addition to overestimating urban areas^38^ (see Methods). Another important limitation of our study is related to the intra-city processes and urban characteristics that cannot be accounted for only by population and area (or density). Case studies on this subject have shown that the urban form and intra-city population distribution have a substantial impact on urban emissions, particularly on transportation emissions. Cities from rapid developing countries such as China and India have undergone through a remarkable decentralization and suburban growth processes^39^. These more dispersed urban forms and the consequent increase of the population living in urban frontier areas have direct implications for commuting and contribute to increasing CO_2_ emissions^39–41^. Regarding this aspect, it would be very interesting for future works to include possible covariates able to account for population imbalance and urban form in our models and thus quantify their impact in a large scale study.

Despite of these limitations, our work adds to the current understanding about the role of urbanization on CO_2_ emissions, shedding light particularly upon the role of population and urban area (including their interactions) on urban emissions. Such interactions are completely overlooked within the urban scaling and per capita density scaling approaches and our work demonstrates that they play an important role in the description of urban emissions. Finally, our framework can be directly applied to other urban metrics in the place of emissions, opening thus a considerable range of possibilities for investigating the interplay between population and area (or population and density) over other important urban metrics.

## Methods

### Dataset

Our dataset is the same as analyzed by Gudipudi et al.^15^ and comprises the CO_2_ emissions from urban areas of the US in the year 2000. As described by Gudipudi et al., this dataset is compiled from different sources through the following steps. First, gridded population data are obtained from the Global Rural-Urban Mapping Project (GRUMP)^42^ and the Global Land Cover Dataset (GLC)^43^. Both datasets are from the year 2000 and are available at a spatial grid resolution of 1 km × 1 km. The GRUMP data are spatially overlaid to the GLC in order to attribute population to the land use, which is classified as urban and non-urban. Next, sectoral emissions data (building and transportation) are obtained from the Vulcan Project^44^. This dataset is initially available at a resolution of 10 km × 10 km and has been down-scaled to 1 km × 1 km to be superimposed on the populated settlements. This process consists of equally splitting the emissions located in a cell of the Vulcan project among all overlapping population cells classified as urban. Finally, the city clustering algorithm (CCA)^27^ is used to systematically define the urban units, leading to the population size (*P* in raw counts), area (*A* in square kilometers), and CO_2_ emissions (*C* in tonnes of CO_2_) for each urban unit. CCA is an iterative clustering algorithm that assigns any two cells to the same cluster if their distance is smaller or equal than a predefined threshold distance *l*.

All results presented in our paper have been obtained using *l* = 5 km, a threshold distance that does not overestimate nor underestimate the urban extents^15^. However, we have verified that our conclusions are very robust against variations in *l* from *l* = 1 km to *l* = 10 km. In particular, the translog model (Eq. (5)) always provides the best description for the US data (Supplementary Fig. 5). We have observed that the scaling exponents *β* and *α* present slightly variations with threshold distance *l* (Supplementary Fig. 6) that have no implications for the conclusions drawn from our findings. The parameters of the Cobb–Douglas (*β*_*P*_ and *β*_*A*_) and translog (*β*_*P*_, *β*_*A*_, and *β*_*C*_) models present somewhat larger variations (Supplementary Fig. 7), but all remain statistically significant, particularly the parameter related to the interaction term in the translog model (*β*_*C*_). These changes affect our point estimates for the Cobb–Douglas (Supplementary Fig. 8) and translog (Supplementary Fig. 9) models. In general, changes in emissions associated with a 1% increase in *P* and *A* tend to increase with *l*. Similarly, the reduction in emissions associated with −1% change in *P* with fixed density or with a 1% change in density with fixed *P* also increase with *l*. This dependence on *l* is smaller in the translog than in the Cobb–Douglas model. The critical product Ω* displays a decreasing trend with *l* (Supplementary Fig. 10), that in turn affects the point from which the effect of *P* and *A* on *C* changes from decreasing to increasing returns to scale. The critical value A* is approximately independent of *l* (Supplementary Fig. 10), indicating that changes in population have more impact than changes in density on emissions regardless the value of *l*. We have further verified that the contour plots of the translog function (Eq. (5)) are very similar for different values of *l* (Supplementary Fig. 11).

These robustness tests are important because there has been a great debate about how to accurately define the correct boundaries of a city^37^. In spite of that, there is still no consensus on this issue nor has a fail-safe procedure for defining the correct boundaries of a city been proposed yet. This issue also has great similarity with the more general concept of clustering and a quite similar issue arises when applying community detection algorithms in complex networks. All these topics have been exhaustively studied, but no silver bullet method exists. In the case of cities, additional complexity emerges because some urban indicators are more spatially constrained than others, and also because people commute to work and move from place to place in the long run.

Partly because of the seminal works by Bettencourt et al.^21^, the use of functional definitions for cities such as the metropolitan statistical areas (MSAs) in US or larger urban units (LUZ) and metropolitan areas (MAs) in Europe have become very popular. These definitions are based on the idea of integrated socio-economic units and appear to be the gold standard for the urban scaling hypothesis as well as other purposes. In particular, MSAs are defined by a core county (or even more than one) having at least 50,000 people aggregated with adjacent counties that display a high degree of interaction (social and economic) with the central county (as measured by commuting flows). While this definition may work well for studying urban scaling, it is very problematic in our case. Since MSAs are made up of counties, they often include vast rural areas which in turn hugely overestimate the urban extent areas. In addition to that, MSAs can also fragment urban clusters into different pieces.

These problems are the main reason why we have chosen the CCA to define the urban units in our study. It is worth noticing that the CO_2_ emissions we have analyzed are from building and transportation, and thus primarily associated with settlements where people reside and commute. Because of that, we argue that the approach of combining gridded data from the Vulcan project, GLC and GRUMP with CCA (proposed in ref. ^15^) provides more precise emission estimates and associated spatial extents of urban clusters. Despite these problems and limitations, we have also applied our models to emissions data associated with MSAs. To do so, we have used the dataset provided by Fragkias et al.^8^ and considered the emissions from the year 2000 (the same used in our analysis). Indeed, these data allowed us to consider not only MSAs but also micropolitan areas (*μ*SAs are defined as labor market areas with population between 10,000 and 50,000 people that are also made up of counties) and both together (core-based statistical areas—CBSAs).

Supplementary Figure 12 shows the urban and per capita density scaling laws for MSAs. We notice that the quality of these relationships is not comparable with those reported in Fig. 1, and a similar situation happens for *μ*SAs and CBSAs. Supplementary Fig. 13A compares the scaling exponents obtained from MSAs, *μ*SAs, and CBSAs with those obtained via CCA with *l* = 5 km. We observe that the values of *β* estimated from these functional city definitions are much closer to one (in agreement with Fragkias et al.^8^) than the values obtained with the CCA approach. As discussed in more detail by Bettencourt et al.^45^, the disaggregation from the true urban unit can either introduce a positive or negative bias in the estimates of *β*. On the other hand, the aggregation of different true urban units tends to make *β* closer to one. In the case of MSAs, it is likely that both disaggregation and aggregation effects play some role, but the fact that *β* is smaller for *μ*SAs than MSAs suggests that aggregation may have greater influence. Due to the poor quality of the per capita density scaling, it is hard to directly compare the values of *α* obtained from MSAs, *μ*SAs, and CBSAs with those estimated with the CCA; however, Supplementary Fig. 13B shows that at least they have the same sign.

We have also applied the models of Eqs. (3) and (5) to MSAs, *μ*SAs, and CBSAs data. Supplementary Fig. 14 shows that these models do not represent an improved description when compared with Eq. (2). For CBSAs and MSAs, Eq. (3) has about the same predictive power as Eq. (2). Supplementary Fig. 15 compares the exponents *β*_*P*_ and *β*_*A*_ obtained from MSAs, *μ*SAs and CBSAs with the CCA values for *l* = 5 km. We notice that *β*_*P*_ is not so different from *β* for these functional city definitions, indicating that the confounding effect of the area is much weaker when compared with the CCA results. This fact reinforces the idea that MSAs and *μ*SAs areas are not good predictors for CO_2_ emissions. We also observe that *β*_*P*_ is much larger for the functional definitions, and that *β*_*A*_ is smaller than the values obtained from the CCA approach. In spite of all discrepancies, the results for MSAs, *μ*SAs, and CBSAs also indicate that population has more impact on emissions than density because |*β*_*P*_ + *β*_*A*_| > |*β*_*A*_|. On the other hand, *β*_*P*_ + *β*_*A*_ ≈ 1 for the functional definitions, while *β*_*P*_ + *β*_*A*_ < 1 for the CCA approach. The approximate constant returns to scale observed for the functional city definitions is also likely to be related to disaggregation and aggregation effects that we previously discussed.

Finally, it is worth remarking that the CCA is also likely to suffer from disaggregation or aggregation effects, as is the case of administrative or functional city definitions. However, differently from such ad hoc definitions, CCA allows us to quantify the impact of such effects by changing the threshold distance *l* and to verify that our conclusions are robust under different values of *l*.

### Cobb–Douglas and the urban scaling models

To relate the Cobb–Douglas model (Eq. (3)) with the urban scaling (Eq. (1)) and the per capita density scaling (Eq. (2)), we first rewrite Eq. (1) as

8 $$C\sim P^\beta = P^{\beta _1}P^{\beta _2},$$

where *β* = *β*_1_ + *β*_2_ stands for the same urban scaling exponent as in Eq. (1). Now, we replace the right-most *P* in the previous equation by the allometry relation between population and area^24,25^, *P* ~ *A*^*δ*^ (where *δ* is another power-law exponent), leading to

9 $$C\sim P^{\beta _1}A^{\beta _2\delta },$$

which has the same form as Eq. (3), that is, *β*_1_ = *β*_*P*_ and *β*_2_*δ* = *β*_*A*_. Consequently,

10 $$\beta = \beta _P + \frac{{\beta _A}}{\delta },$$

and the two approaches are uniquely related only if there is another constraint for the exponents. One possibility is by imposing constant returns to scale in the Cobb–Douglas model (*β*_*A*_ + *β*_*P*_ = 1), which leads to

11 $$\beta _P = \frac{{\beta \delta - 1}}{{\delta - 1}}\,{\mathrm{and}}\,\beta _A = \frac{{\delta (\beta - 1)}}{{1 - \delta }},$$

for *δ* ≠ 1; while for *δ* = 1, we obtain *β*_*P*_ = 1 and *β*_*A*_ = 0. Thus, the existence of an additional constraint for the parameters *β*_*P*_ and *β*_*A*_ implies that the Cobb–Douglas model is equivalent to Eqs. (1) and (2); otherwise, it represents a generalization.

The Cobb–Douglas model with *β*_*A*_ + *β*_*P*_ = 1 is also related to scaling relationships between indicator density and population density^46–48^. To obtain this connection, we rewrite Eq. (3) as

12 $$C\sim P^\theta A^{1 - \theta },$$

where *β*_*P*_ = *θ* and *β*_*A*_ = 1 − *θ*. Next, we divide both sides by *A*

13 $$C/A\sim (P/A)^\theta ,$$

leading to a scaling relationship between CO_2_ density and population density.

### Analogy with the production functions

As we have argued, our approach is inspired by the economic theory of production functions^26^. By following this analogy, we have considered the urban emissions as the output and population and area (or density) as the inputs of a two-factor production process mediated by cities. The mathematical formula that describes the possible relations between the inputs (*P* and *A*) and the output (*C*) is the production function, that is, *C* = *F*(*P*,*A*). The functional forms for *F* used in our work comprise the most widely known and used production functions in economics and should be viewed as an empirical/phenomenological description (as it also happens in economics). In what follows, we summarize concepts from the economic theory of production functions that have been used in our work.

*Elasticity of scale*. The elasticity of scale *ε* is the ratio between a proportionate change in the output (emissions) and a proportionate change in the inputs (population and area), that is, *ε* = (*dF*/*F*)/(*dP*/*P*), where *dP*/*P* = *dA*/*A* represents the proportionate change in the inputs. This measure quantifies the impact of changing population and area on emissions.

*Technical rate of substitution*. The technical rate of substitution measures the rate at which an input must change in response to a change in the other input so that the output remains constant. In absolute value, it represents the slope of the isoquants of the production function. For instance, assuming a particular value for the output *F*(*P*, *A*) = *C*_0_, the technical rate of substitution between area and population is $\delta _P^A = dA/dP$.

*Elasticity of substitution*. The elasticity of substitution *σ* somehow summarizes the shape of an isoquant. It is defined as the ratio between a proportionate change in the inputs and the associated proportionate change in the slope of the isoquant. Mathematically, we write $\sigma = \frac{{[d(P/A)/(P/A)]}}{{[d(dP/dA)/(dP/dA)]}} = \frac{{d\,{\mathrm{log}}\,(P/A)}}{{d\,{\mathrm{log}}\,(dP/dA)}}$, where the numerator represents the proportionate change in the inputs and the denominator the proportionate change in the slope of the isoquant. This measure quantifies the efficiency at which population and area substitute each other.

*Marginal products*. The marginal product of an input is defined as the (infinitesimal) change in the output resulting from a (infinitesimal) change in one of the inputs. For instance, the marginal product of population is Δ_*P*_ = *dF*/*dP* (it can also be defined in terms of logarithmic quantities: Δ_*P*_  = *d* log *F*/*d* log *P*).

We summarize all these properties calculated for Cobb–Douglas (Eq. (3)) and translog (Eq. (5)) models in Supplementary Table 2.

### Fitting models with the ridge regression approach

As we have discussed in the main text, multicollinearity is present in the models of Eqs. (3) and (5). This effect happens when at least two predictors in a multiple linear regression are correlated to each other^29,30^. Under this situation and depending on the degree of correlation among the predictors, ordinary-least-squares estimates of the parameters can be unstable against minor changes in the input data and also display large standard errors. To better illustrate this problem, consider the simple linear model

14 $$y\sim a_1x_1 + a_2x_2,$$

where *y* is the response variable, *x*_1_ and *x*_2_ are the predictors, and *a*_1_ and *a*_2_ are the linear coefficients. The least-squares estimator for the parameters is usually written as $${\boldsymbol{a}} = \left[ {\begin{array}{*{20}{l}} {a_1} \hfill \\ {a_2} \hfill \end{array}} \right] = ({\boldsymbol{X}}^T{\boldsymbol{X}})^{ - 1}{\boldsymbol{X}}^T{\boldsymbol{y}}$$, where $${\boldsymbol{y}} = \left[ {\begin{array}{*{20}{c}} {y_1^{(1)}} \\ \vdots \\ {y^{(n)}} \end{array}} \right]$$ is an *n* × 1 vector of the response variables, $${\boldsymbol{X}} = \left[ {\begin{array}{*{20}{c}} {x_1^{(1)}} & {x_2^{(1)}} \\ \vdots & \vdots \\ {x_1^{(n)}} & {x_1^{(n)}} \end{array}} \right]$$ is an *n* × 2 matrix of the regressors, and *n* is the number of observations. If the values of predictors are strongly correlated, the inversion of the matrix ***X***^*T*^***X*** can become unstable, and consequently lead to unstable estimates for the linear coefficients.

To account for the multicollinearity problem, we have fitted Eqs. (3) and (5) by using the ridge regression approach^29,30^. This method solves the matrix inversion problem by adding a constant *λ* to the diagonal elements of ***X***^*T*^***X***, so that the ridge estimator for the linear coefficients is ***a*** = (***X***^*T*^***X*** + *λ****I***)^−1^***X***^*T*^***y***, where ***I*** is the identity matrix. The ridge estimation is equivalent to finding the optimal linear coefficients that minimize the residual sum of squares plus a penalty term (also called regularization parameter) proportional to the sum of the squares of the linear coefficients^29,30^, that is, finding the ***a*** that minimizes the objective function ∥***y*** − ***Xa***∥^2^ + *λ*∥***a***∥^2^. The optimal value of *λ* is usually unknown in practice and needs to be estimated from data. To do so, we have used the approach of searching for the value of *λ* that minimizes the mean squared error (MSE) in a leave-one-out cross validation strategy. In this approach, we estimate ***a*** (for a given *λ*) using all data except for one point that is used for calculating the squared error. This process is repeated until every data point is used exactly once for estimating the squared error, and then we calculate the value of the MSE for a given *λ*. The optimal value of *λ* = λ* is the one that minimizes the average value of the MSE estimated with the leave-one-out cross validation method. We have also standardized all predictors before searching for the optimal value λ*. This is a common practice when dealing with regularization methods and ensures that the penalty term is uniformly applied to the predictors, that is, the normalization makes the scale of the predictors comparable and prevents variables with distinct ranges from having uneven penalization.

The standardized version of Eq. (3) can be written as

15 $${\mathrm{log}}\,C\sim \tilde \beta _P\widehat {{\mathrm{log}}\,P} + \tilde \beta _A\widehat {{\mathrm{log}}\,A},$$

where

16 $$\widehat {{\mathrm{log}}\,P} = \frac{{{\mathrm{log}}\,P - \mu _P}}{{\sigma _P}}\,{\mathrm{and}}\,\widehat {{\mathrm{log}}\,A} = \frac{{{\mathrm{log}}\,A - \mu _A}}{{\sigma _A}}.$$

In addition, *μ*_*P*_ is the average value of log*P* $\left( {\mu _P = \frac{1}{n}{\sum} {{\mathrm{log}}} P} \right)$, *σ*_*P*_ is the standard deviation of log*P* $\left[ {\sigma _P^2 = \frac{1}{{n - 1}}{\sum} {({\mathrm{log}}P - \mu _P)^2} } \right]$, *μ*_*A*_ is the average value of log *A* $\left( {\mu _A = \frac{1}{n}{\sum} {{\mathrm{log}}} A} \right)$, and *σ*_*A*_ is the standard deviation of log*A* $\left[ {\sigma _A^2 = \frac{1}{{n - 1}}{\sum} {({\mathrm{log}}\,A - \mu _A)^2} } \right]$. It is worth remarking that the values of $\widehat {{\mathrm{log}}\,P}$ and $\widehat {{\mathrm{log}}\,A}$ are invariant against changes in the scale of *P* and *A*, that is, their values do not change under the transformations *P* → *νP* and *A* → *νA*, where *ν* is a positive constant. The same invariance holds for *σ*_*P*_ and *σ*_*A*_, whereas the average values change according to *μ*_*P*_ → log *ν* + *μ*_*P*_ and *μ*_*A*_ → log *ν* + *μ*_*A*_.

The connection between the parameters of the standardized model ($\tilde \beta _P$ and $\tilde \beta _A$) and the usual ones (*β*_*P*_ and *β*_*A*_) is obtained by plugging Eq. (16) into Eq. (15), collecting the terms multiplying log *P* and log *A*, and then directly comparing the results with Eq. (3). By following this approach, we find that

17 $$\beta _P = \frac{{\tilde \beta _P}}{{\sigma _P\,\sigma _A}}\,{\mathrm{and}}\,\beta _A = \frac{{\tilde \beta _A}}{{\sigma _P\,\sigma _A}},$$

where we observe that *β*_*P*_ and *β*_*A*_ are independent of the units of *P* and *A* (as they should be, since *σ*_*P*_ and *σ*_*A*_ are scale invariants). Supplementary Fig. 16 illustrates how the ridge regression approach is applied to the model of Eq. (15). Supplementary Fig. 16A shows the dependence of the MSE on the values of *λ*, from which we obtain λ* = 9.78. Supplementary Fig. 16B shows the dependence of the parameters $\tilde \beta _A$ and $\tilde \beta _P$, whose values for the optimal regularization term $\left( {\lambda = \lambda ^ \ast } \right)$ are $\tilde \beta _P = 0.37 \pm 0.02$ and $\tilde \beta _A = 0.24 \pm 0.02$, which correspond to *β*_*P*_ = 0.31 ± 0.01 and *β*_*A*_ = 0.45 ± 0.03. The errors in $\tilde \beta _P$ and $\tilde \beta _A$ stand for the standard deviation of their values estimated over 1000 random samples with replacement of the data, as shown in Supplementary Fig. 17. The errors in *β*_*P*_ and *β*_*A*_ are calculated with common error propagation formulas. Moreover, the *p*-values of permutation tests reject the null hypothesis that these parameters are equal to zero.

In the case of Eq. (5), its standardized version can be rewritten as

18 $${\mathrm{log}}\,C\sim \tilde \beta _P\,\widehat {{\mathrm{log}}\,P} + \tilde \beta _A\,\widehat {{\mathrm{log}}\,A} + \tilde \beta _C\,\widehat {{\mathrm{log}}\,(P)}\,\widehat {{\mathrm{log}}\,(A)},$$

where the connecting formulas

19 $$\beta _P = \frac{{\sigma _A\tilde \beta _P - \mu _A\tilde \beta _C}}{{\sigma _P\,\sigma _A}},\beta _A = \frac{{\sigma _P\tilde \beta _A - \mu _P\tilde \beta _C}}{{\sigma _P\,\sigma _A}},{\mathrm{and}}\,\beta _C = \frac{{\tilde \beta _C}}{{\sigma _P\,\sigma _A}},$$

are obtained as in the previous case. Unlike the models of Eqs. (1)–(3), the generalization expressed by Eq. (5) is not scale-invariant and thus its parameters depend on the measurement units. In particular, if the area is rescaled by a factor *ν* (*A* → *νA*), *β*_*P*_ is incremented by the factor −*β*_*C*_ log *ν* and *β*_*A*_ remains unchanged. Similarly, if population is rescaled by *ν* (*P* → *νP*), *β*_*A*_ is also incremented by −*β*_*C*_ log *ν* and *β*_*P*_ remains unchanged. Only *β*_*C*_ is invariant against scale changes in *P* and *A*. Thus, all interpretations related to the behavior of the emissions obtained from the model Eq. (5) involve the assumption that area is expressed in units of km^2^ and raw population counts. Supplementary Fig. 18 illustrates how the ridge regression approach is applied to the model of Eq. (18). Supplementary Fig. 18A shows the dependence of the MSE on *λ*, from which λ* = 8.67 is obtained. Supplementary Fig. 18B shows the behavior of the model parameters as a function of *λ*. This approach yields $\tilde \beta _P = 0.40 \pm 0.02$, $\tilde \beta _A = 0.17 \pm 0.02$, and $\tilde \beta _C = 0.044 \pm 0.006$ for *λ* = λ*, which correspond to *β*_*P*_ = 0.28 ± 0.02, *β*_*A*_ = 0.14 ± 0.05, and *β*_*C*_ = 0.07 ± 0.01. The standard errors are calculated as in the previous case and the *p*-values of the permutation tests reject the null hypothesis that the model parameters are equal to zero (Supplementary Fig. 3).

In addition to the models of Eqs. (3) and (5), we have further tested for a more general translog model (full translog model) having quadratic terms in log*P* and log*A*, that is,

20 $$\begin{array}{*{20}{l}} {{\mathrm{log}}\,C} \hfill & {\sim \beta _P{\mathrm{log}}\,P + \beta _A{\mathrm{log}}\,A + \beta _C\,{\mathrm{log}}(P){\mathrm{log}}(A)} \hfill \\ {} \hfill & { + \beta _{P\prime }({\mathrm{log}}\,P)^2 + \beta _{A\prime }({\mathrm{log}}\,A)^2,} \hfill \end{array}$$

where *β*_*P*_*'* and *β*_*A*_*'* are additional parameters. This expression can also be related to the CES production by applying the Taylor series expansion to Eq. (4) around the point *γ* = 0 (the Kmenta approximation^49^). We have fitted Eq. (20) by following the same procedure used for Eqs. (3) and (5). In particular, Supplementary Fig. 19 illustrates how the ridge approach is applied to this model and Supplementary Fig. 20 shows the best fitting parameters and their estimated errors. However, as shown in Supplementary Fig. 2, the full translog model of Eq. (20) does not improve the goodness of the fit when compared with Eq. (5).

## Supplementary information

Supplementary Information

## Data Availability

The dataset used in this study were obtained from Gudipudi et al.^15^, which in turn rely on freely available data obtained from the Global Rural-Urban Mapping Project (GRUMP)^42,^ the Global Land Cover Dataset (GLC)^43^, and the Vulcan Project^44^. All data supporting the findings of this study are available from the corresponding authors on reasonable request.

## Code availability

The code used for analyzing data is available from the corresponding authors on reasonable request.
