# Supplementary material for: Effects of changing population or density on urban carbon dioxide emissions
Source: Nat Commun. 2019 Jul 19;10:3204. doi: 10.1038/s41467-019-11184-y (PMC6642210; doi:10.1038/s41467-019-11184-y)
Supplement: Supplementary file 1 — Supplementary Information [file 41467_2019_11184_MOESM1_ESM.pdf]

Supplementary Information for

Effects of changing population or density on urban carbon dioxide emissions

Haroldo V. Ribeiro et al., Nature Communications, 2019.

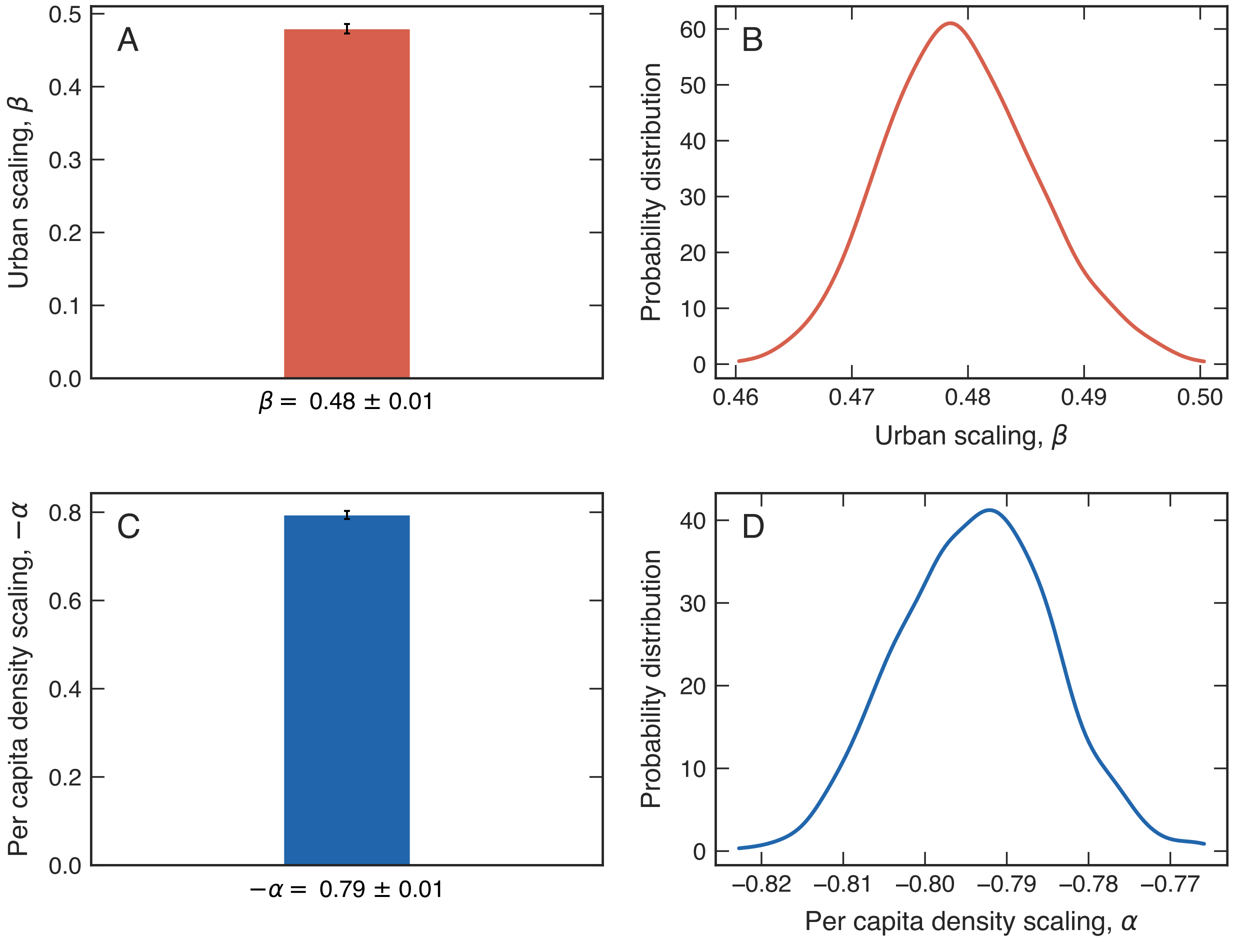

Supplementary Figure 1. **Estimating the errors in the parameters of Eqs. (1) [urban scaling] and (2) [per capita density scaling].** Bar plots in panels (A) and (C) show the average values of the parameters β and α estimated after fitting the models to 1000 random samples (with replacement) of our data. In these panels, error bars stand for the standard deviation. Panels (B) and (D) show the probability distribution of the values of β and α over all random samples. In both cases, the p-values of the permutation test are virtually zero, rejecting the null hypothesis that β and α are zero.

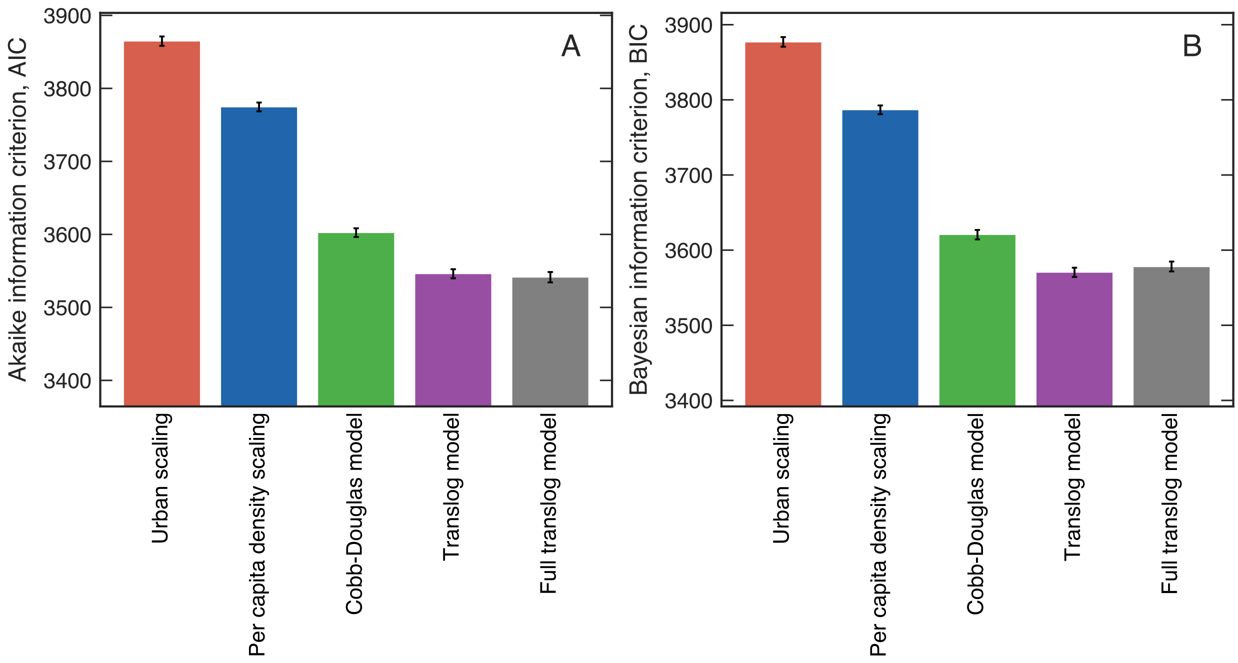

Supplementary Figure 2. **Comparing the goodness of the fit among the models of Eqs. (1) [urban scaling], (2) [per capita density scaling], (3) [Cobb-Douglas model], (5) [translog model], and (20) [full translog model].** (A) Average Akaike information criterion (AIC) and (B) Bayesian information criterion (BIC) values for each model. These values were estimated from 1000 different random samplings (with replacement) of our data. The smaller the values of the AIC and BIC coefficients, the better is the quality of fit. Error bars are 95% bootstrap confidence intervals. We note that the models of Eqs. (5) and (20) provide significantly lower values when compared with all other models; however, no significant difference is observed between these two models. It is worth noting that these coefficients include a penalty term that depends on the number of parameters, allowing a fair comparison among models with different number of parameters. We further tested whether different linear regression fitting approaches improve the AIC and BIC for the urban scaling and per capita density scaling. We find that robust ordinary least squares, total least squares, and least absolute deviations regression do not improve the quality of fits of Eqs. (1) and (2).

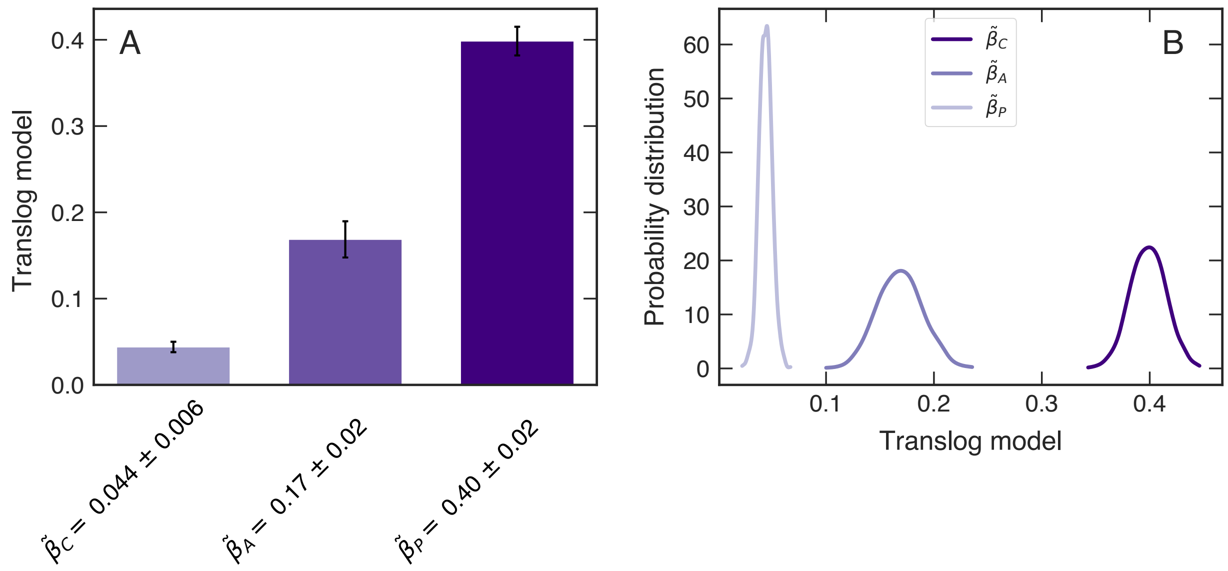

Supplementary Figure 3. **Estimating the errors in the parameters of Eq. (5) [translog model].** (A) The bar plot shows the average values of the parameters $\tilde{\beta}_P$, $\tilde{\beta}_A$, and $\tilde{\beta}_C$ estimated after fitting the model to 1000 random samples (with replacement) of our data. Error bars stand for the standard deviation of these values. (B) Probability distribution of the values of $\tilde{\beta}_P$, $\tilde{\beta}_A$, and $\tilde{\beta}_C$ over all random samples. The permutation test on the model coefficients rejects the null hypothesis that they are equal to zero at the 95% confidence level.

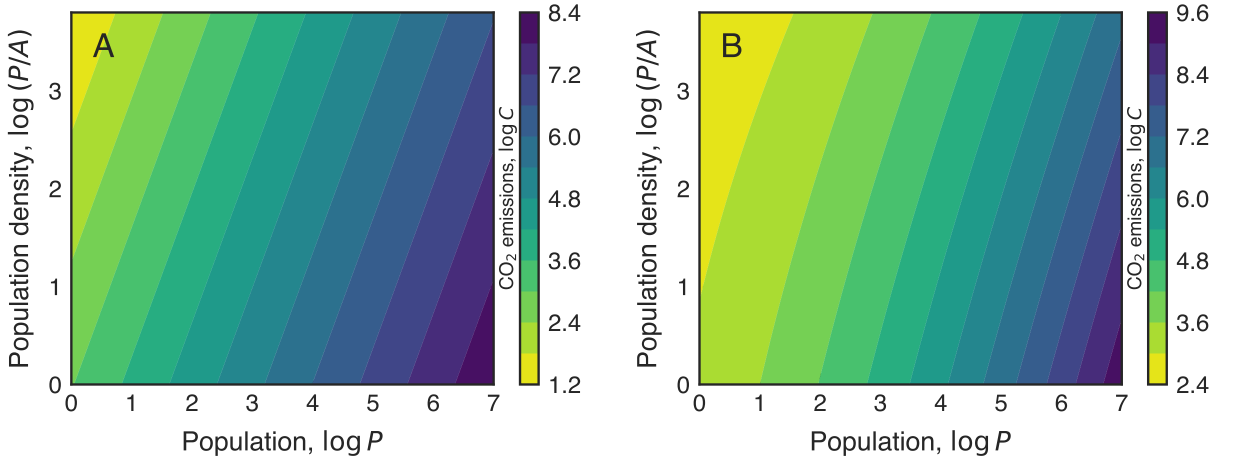

Supplementary Figure 4. **Contour plots of Eqs. (3) [Cobb-Douglas model] and (5) [translog model] in terms of population density.** (A) Contour plot of Eq. (3) rewritten in terms of population density, that is, $C \sim P^{\beta_P+\beta_A}(P/A)^{-\beta_A}$, with $\beta_P = 0.31 \pm 0.01$ and $\beta_A = 0.45 \pm 0.02$. (B) Contour plot of Eq. (5) rewritten in terms of population density, that is, $C \sim P^{\beta_P+\beta_A+\beta_C \log P}(P/A)^{-\beta_A-\beta_C \log P}$, with $\beta_P = 0.28 \pm 0.02$, $\beta_A = 0.14 \pm 0.05$, and $\beta_C = 0.07 \pm 0.01$. We have employed base-10 logarithmic quantities in all panels.

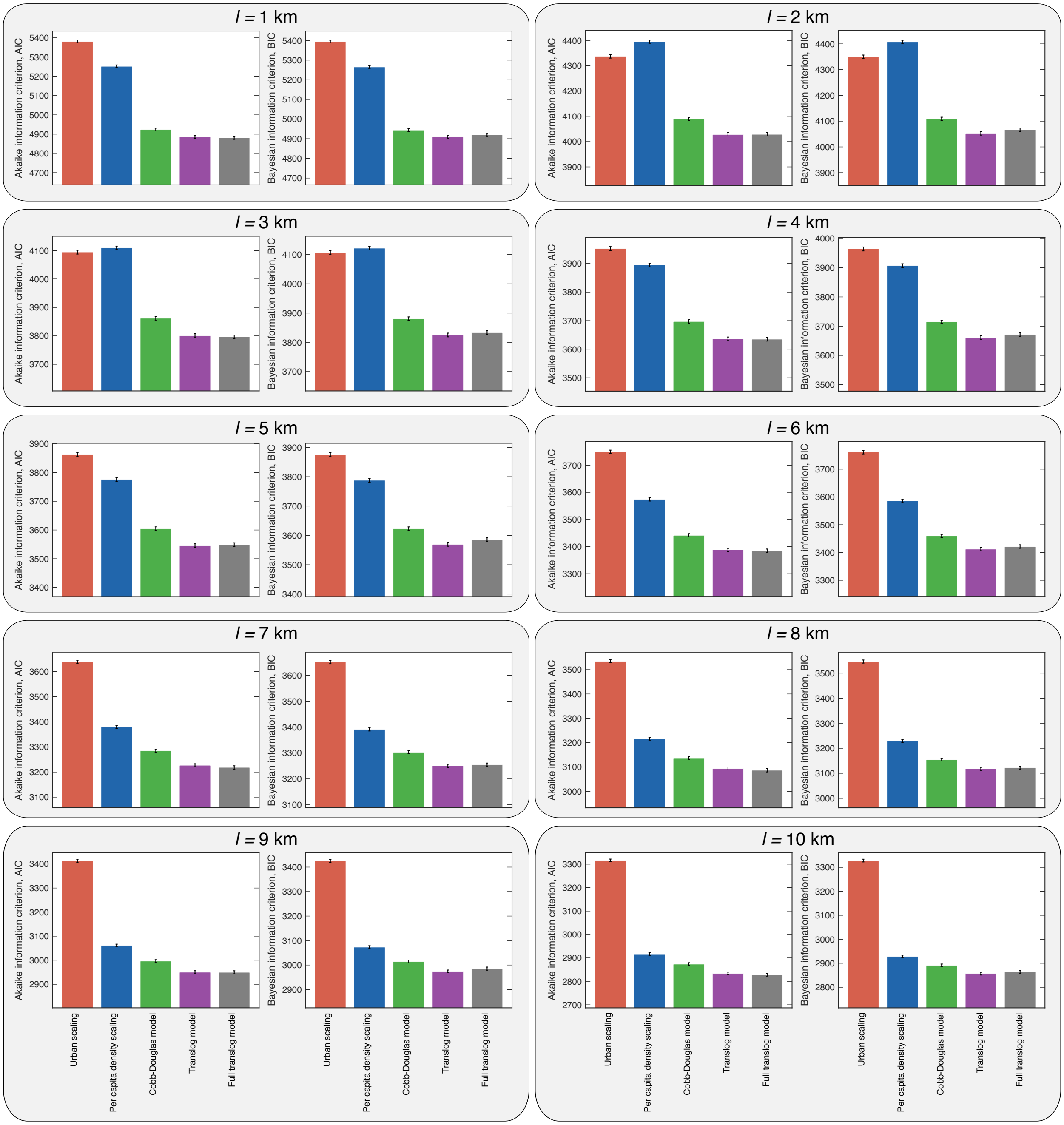

Supplementary Figure 5. **Comparing the goodness of the fit among the models of Eqs. (1) [urban scaling], (2) [per capita density scaling], (3) [Cobb-Douglas model], (5) [translog model], and (20) [full translog model] under different values of the CCA threshold distance l.** Each panel shows the average Akaike information criterion (AIC) and the Bayesian information criterion (BIC) estimated from 1000 different random samplings (with replacement) of our data for different values of $l \in (1, 2, 3, 4, 5, 6, 7, 8, 9, 10)$ km. In all plots, the error bars are 95% bootstrap confidence intervals. We note that the translog model [Eq. (5)] always provides the best description regardless the value of l.

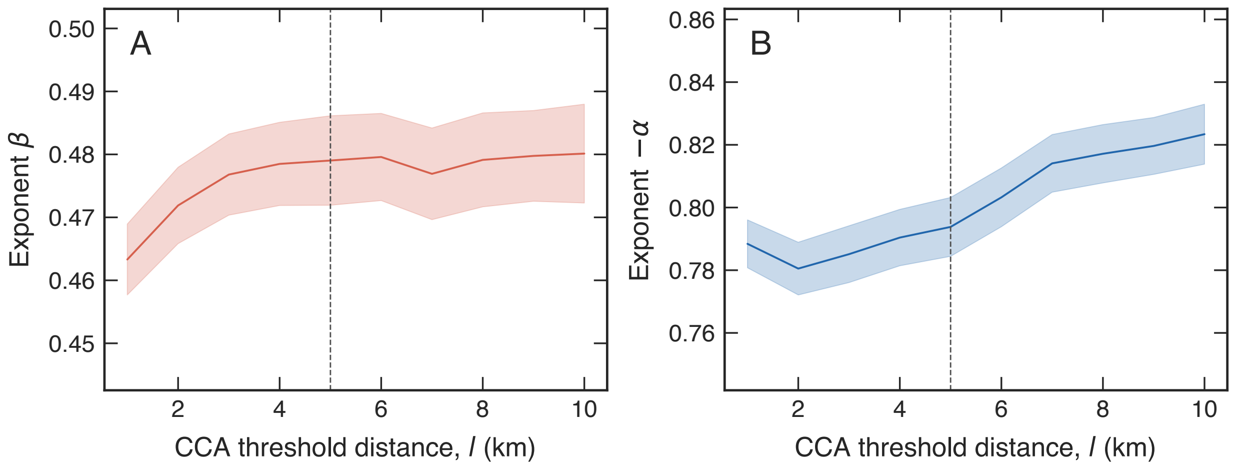

Supplementary Figure 6. **The urban scaling exponents β [Eq. (1), (A)] and α [Eq. (2), (B)] as functions of the CCA threshold distance l.** In both plots, the shaded regions stand for the standard deviation in the parameters estimated after fitting the models to 1000 random samples (with replacement). The vertical lines indicate the values for $l = 5$ km.

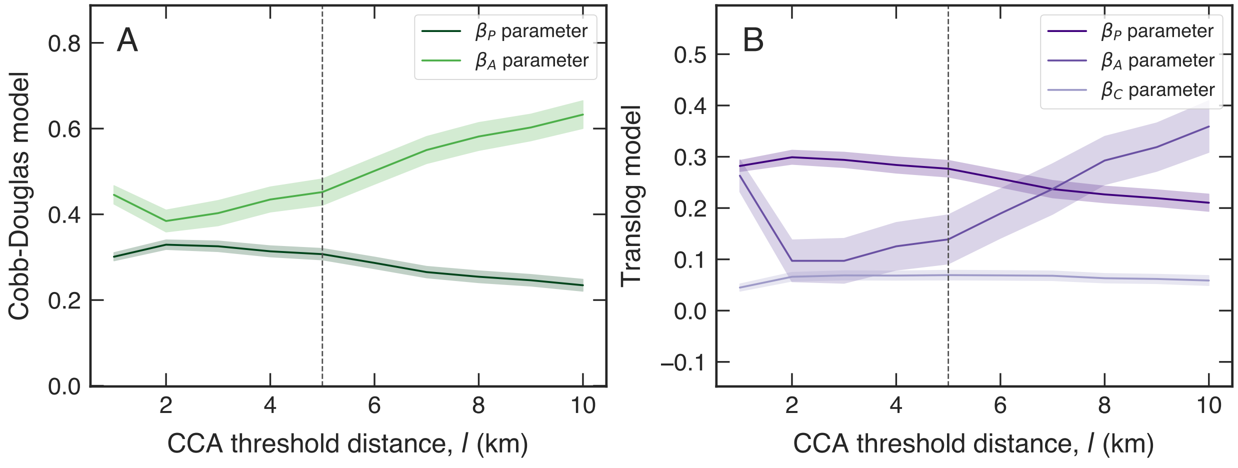

Supplementary Figure 7. **The parameters of Cobb-Douglas [β_P and β_A in Eq. (3), (A)] and translog [β_P, β_A, and β_C in Eq. (5), (B)] models as functions of the CCA threshold distance l.** In both plots, the shaded regions stand for the standard deviation in the parameters estimated after fitting the models to 1000 random samples (with replacement). The vertical lines indicate the values for $l = 5$ km.

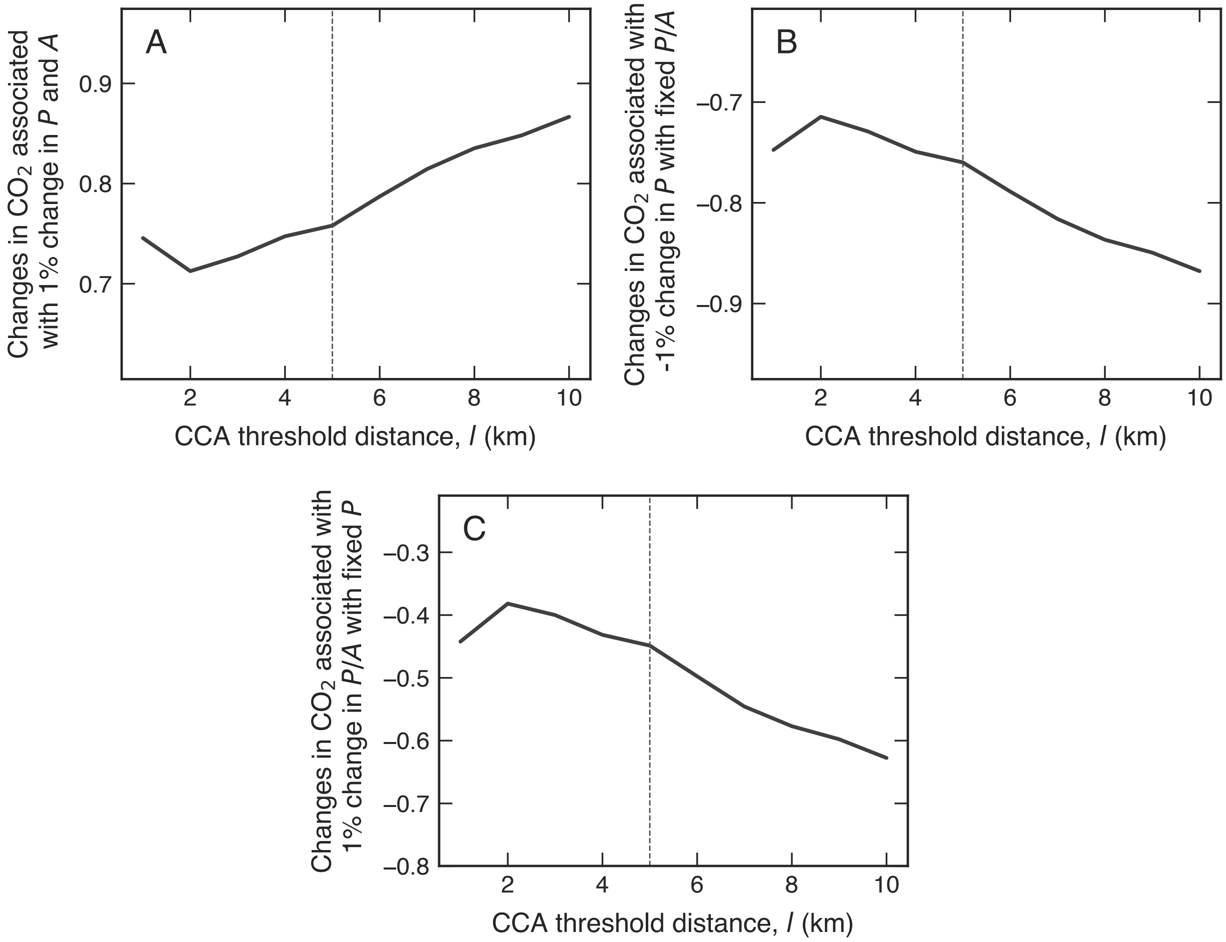

Supplementary Figure 8. **Changes in the point estimates of the Cobb-Douglas model [Eq. (3)] reported in the manuscript against variations in CCA threshold distance l.** (A) The effect of a 1% change in population and area on the emissions as a function of l. (B) The effect of a -1% change in population (with fixed density) on the emissions as a function of l. (C) The effect of a 1% change in population density (with fixed population) on the emissions as a function of l. The vertical lines indicate the values for $l = 5$ km.

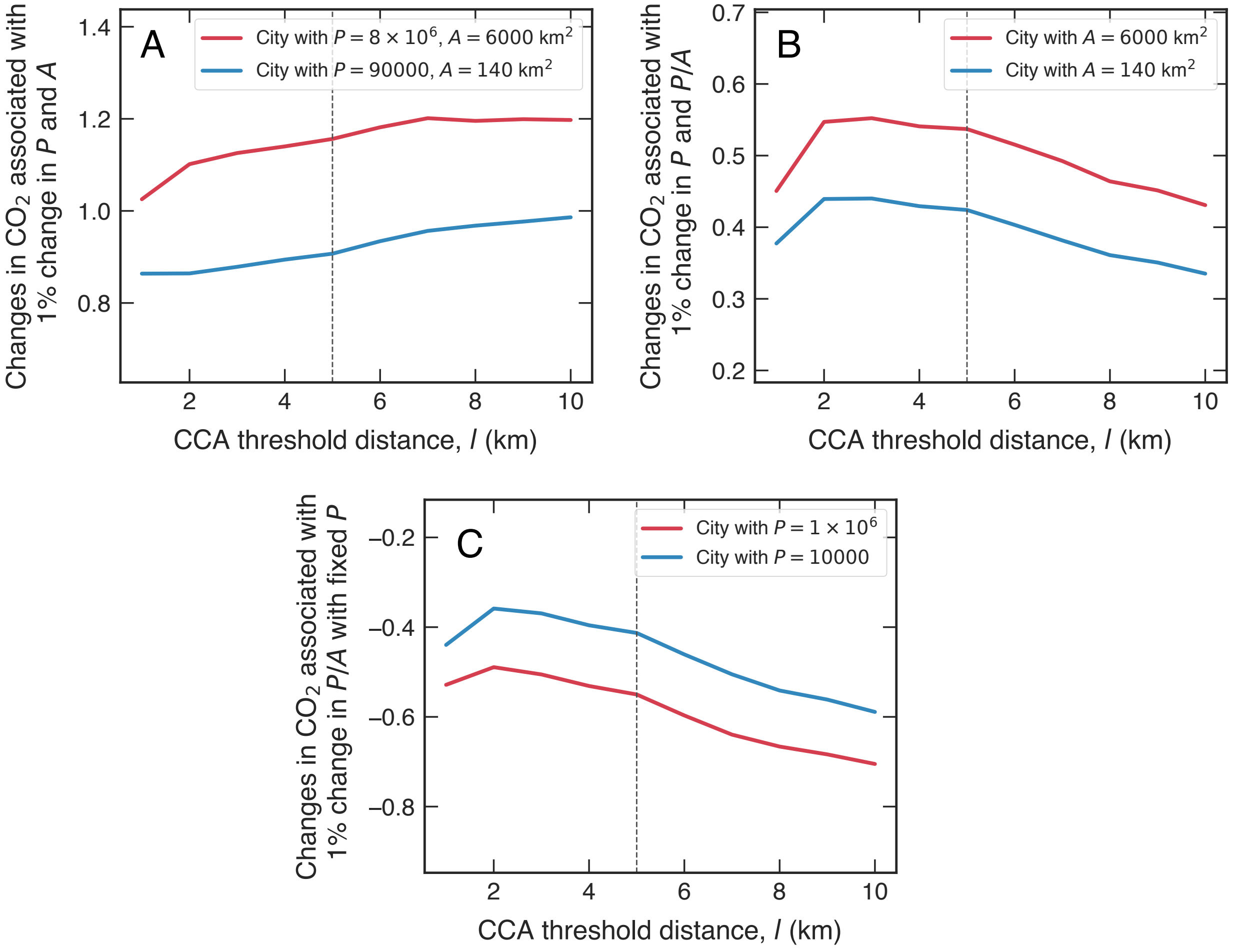

Supplementary Figure 9. **Changes in the point estimates of the translog model [Eq. (5)] reported in the manuscript against variations in the CCA threshold distance l.** (A) The effect of a 1% change in population and area of two hypothetical cities with different initial values for P and A (as indicated within the plot) as function of l. (B) The effect of a 1% change in population (with fixed density) of two hypothetical cities with different initial values for A (as indicated within the plot) as function of l. (C) The effect of a 1% change in population density (with fixed population) of two hypothetical cities with different initial values for P (as indicated within the plot) as function of l. The vertical lines indicate the values for $l = 5$ km.

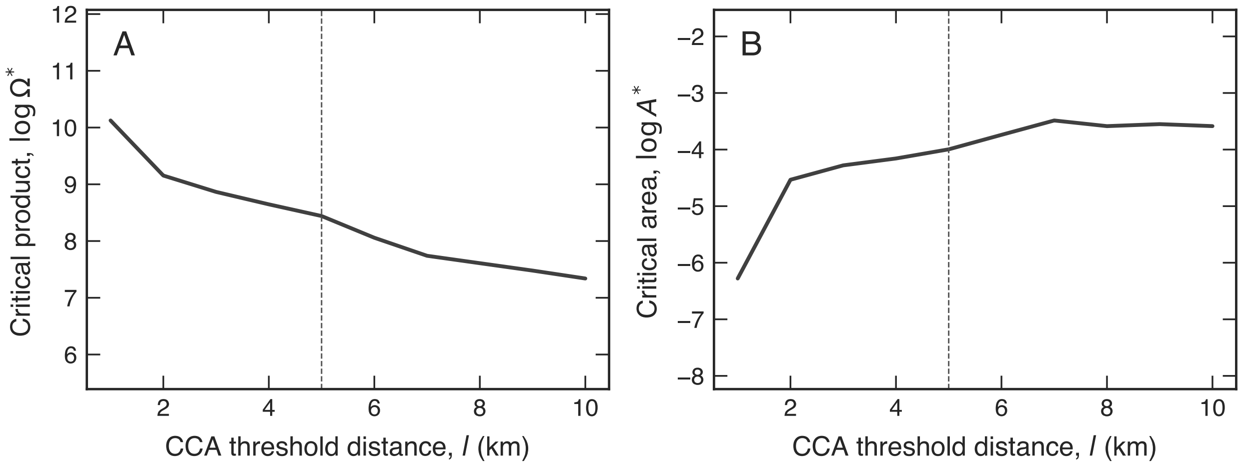

Supplementary Figure 10. **Robustness of the critical values Ω^* and A^* against variations in the CCA threshold distance l.** (A) The critical product $\Omega^* = 10^{(1-\beta_P-\beta_A)/\beta_C}$ and (B) the critical area $A^* = 10^{-\beta_P/\beta_C}$ as a function of l. The vertical lines indicate the values for $l = 5$ km.

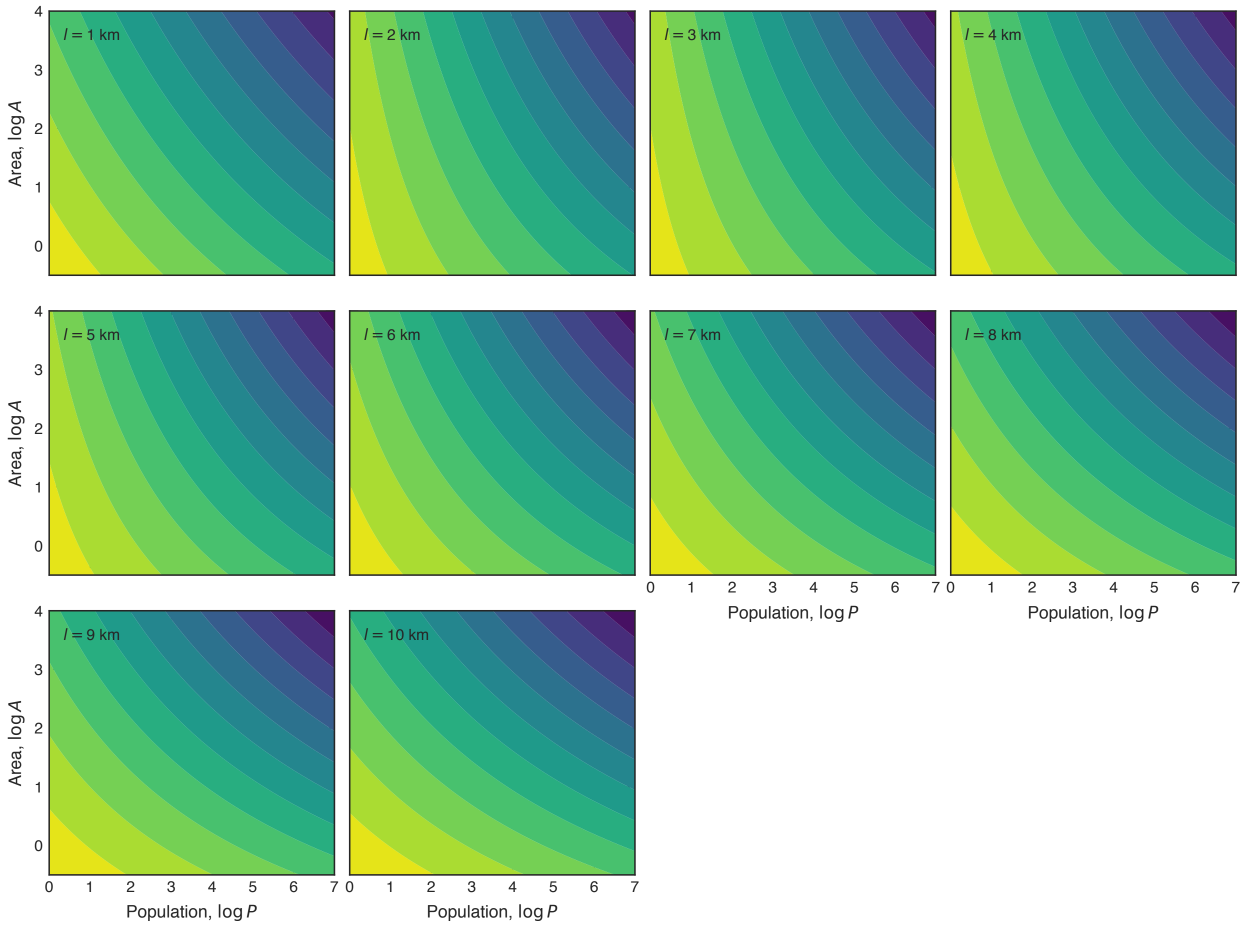

Supplementary Figure 11. **The shape of the isoquants of the translog model Eq. (5)] against variations in the CCA threshold distance l.** Each panel shows the contour plot of the translog model [Eq. (5)] with the best fitting parameters for different values of l (indicated in the plots).

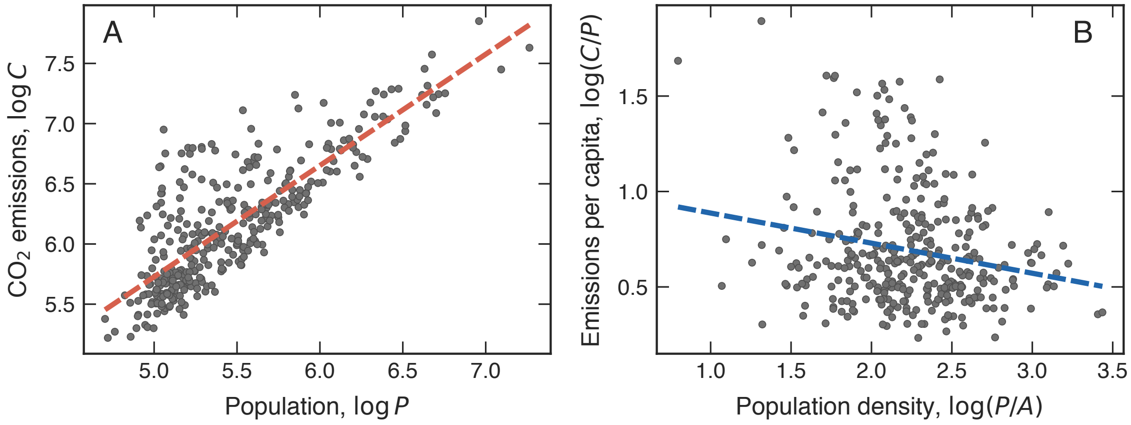

Supplementary Figure 12. **(A) Urban scaling and (B) per capita density scaling applied to Metropolitan Statistical Areas (MSAs)**. In both plots, each dot is associated with a MSA and the dashed lines represents power-law fits [Eq. (1)] with exponents $\beta = 0.92 \pm 0.04$ (panel A) and $\alpha = -0.16 \pm 0.04$ (Panel B). Emissions data were obtained from Ref.[8].

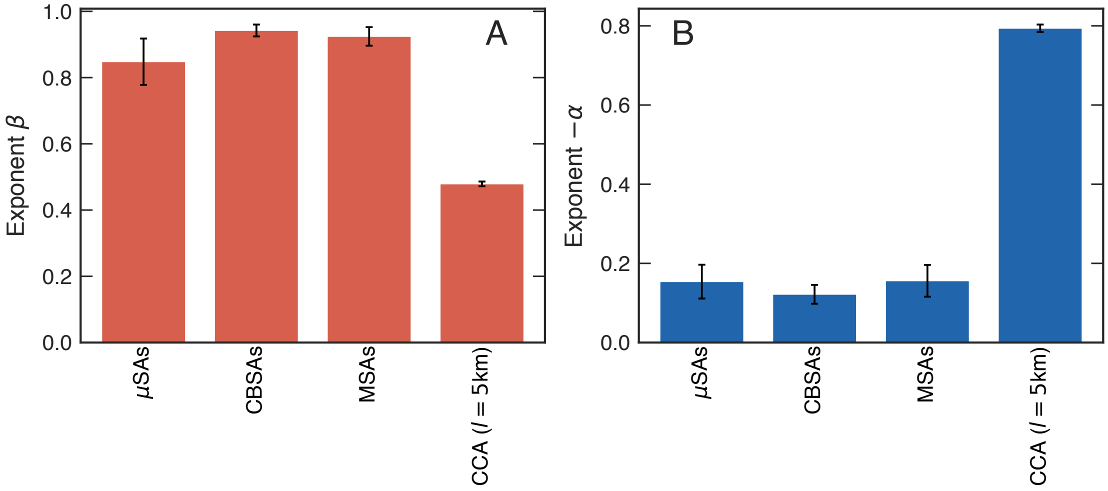

Supplementary Figure 13. **Comparison among the exponents (A) β and (B) α obtained from Metropolitan Statistical Areas (MSAs), Micropolitan Areas (μSAs), and Core Based Statistical Areas (CBSAs) data with those estimated via City Clustering Algorithm (CCA with $l = 5$ km).** Bar plots in panels (A) and (B) show the average values of the parameters β and α estimated after fitting the models to 1000 random samples (with replacement) of data for each definition of city. Error bars stand for the standard deviation of these values.

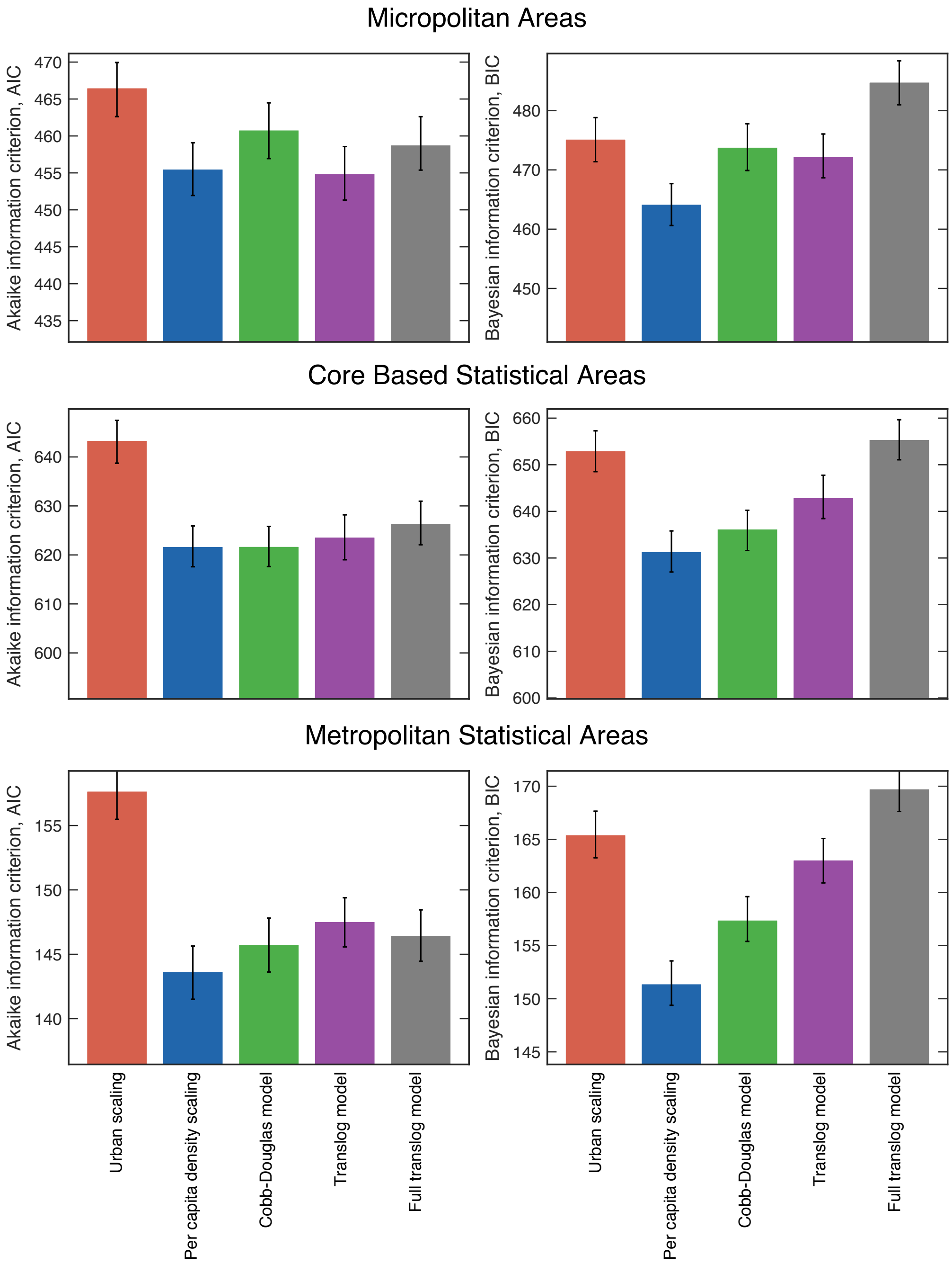

Supplementary Figure 14. **Comparing the goodness of the fit among the models of Eqs. (1) [urban scaling], (2) [per capita density scaling], (3) [Cobb-Douglas model], (5) [translog model], and (20) [full translog model] under Metropolitan Statistical Areas (MSAs), Micropolitan Areas (μSAs), and Core Based Statistical Areas (CBSAs) data.** Each panel shows the average Akaike information criterion (AIC) and the Bayesian information criterion (BIC) estimated from 1000 different random samplings (with replacement) of data for each definition of city. In all plots, the error bars are 95% bootstrap confidence intervals.

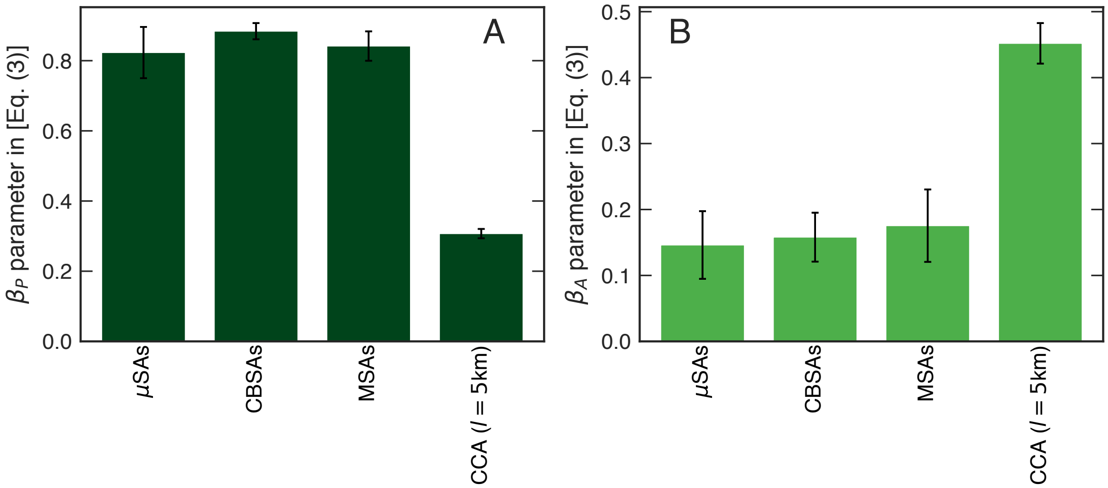

Supplementary Figure 15. **Comparison among the Cobb-Douglas exponents [Eq. (3)] obtained from Metropolitan Statistical Areas (MSAs), Micropolitan Areas (μSAs), and Core Based Statistical Areas (CBSAs) data with those estimated via City Clustering Algorithm (CCA with l = 5 km).** Bar plots in panels (A) and (B) show the average values of the parameters β_P and β_A estimated after fitting the models to 1000 random samples (with replacement) of data for each definition of city. Error bars stand for the standard deviation of these values.

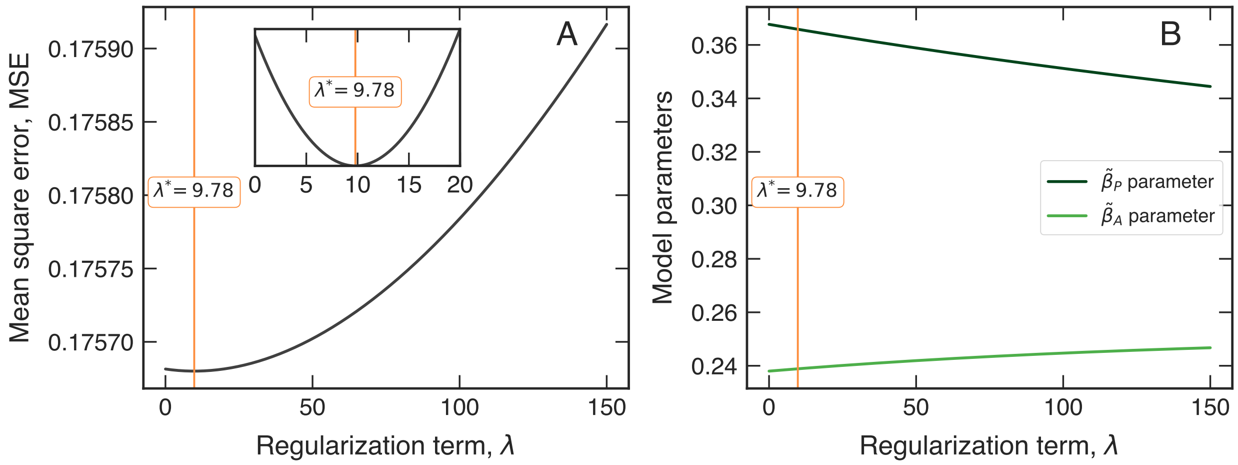

Supplementary Figure 16. **Fitting Eq. (3) [Cobb-Douglas model] to data with the ridge regression approach.** (A) The black curve shows the dependence of the mean square error (MSE) on the regularization term (λ). The average value is estimated using a leave-one-out cross validation strategy. The vertical line indicates the minimum value of the MSE occurring at $\lambda = \lambda^* = 9.78$. The inset highlights the behavior around the minimum. (B) Dependence of the parameters $\tilde{\beta}_P$ and $\tilde{\beta}_A$ on the regularization term (λ). The optimal values for $\lambda = \lambda^*$ are $\tilde{\beta}_P = 0.37 \pm 0.02$ and $\tilde{\beta}_A = 0.24 \pm 0.02$.

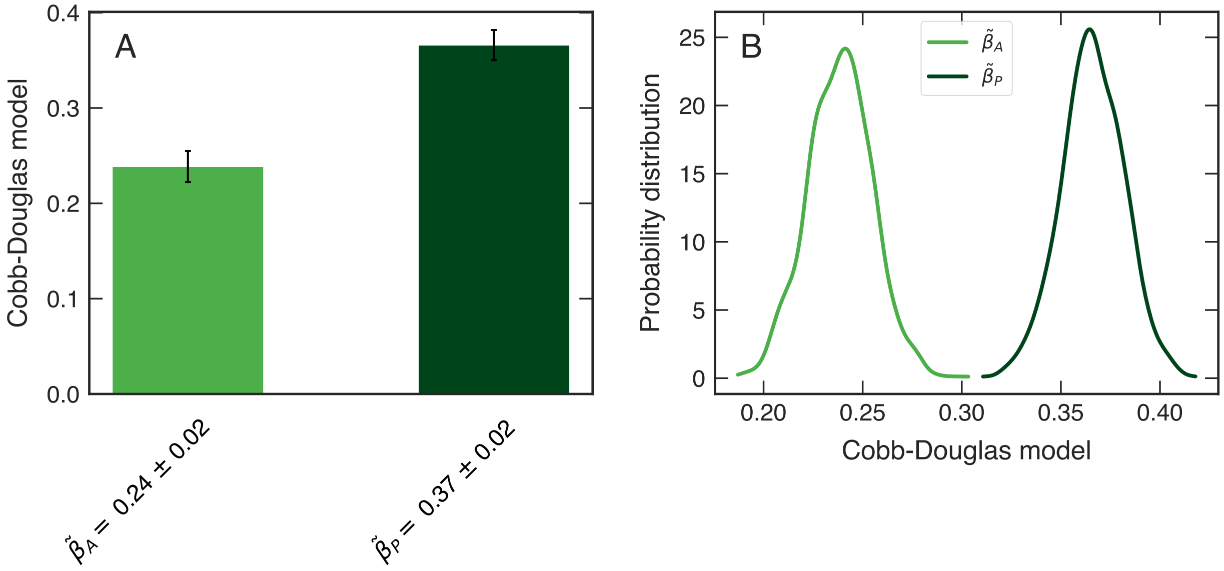

Supplementary Figure 17. **Estimating the errors in the parameters of Eq. (3) [Cobb-Douglas model].** (A) The bar plot shows the average values of the parameters $\tilde{\beta}_P$ and $\tilde{\beta}_A$ estimated after fitting the model to 1000 random samples (with replacement) of our data. Error bars stand for the standard deviation of these values. (B) Probability distribution of the values of $\tilde{\beta}_P$ and $\tilde{\beta}_A$ over all random samples. The permutation test on the model coefficients rejects the null hypothesis that they are equal to zero at the 95% confidence level.

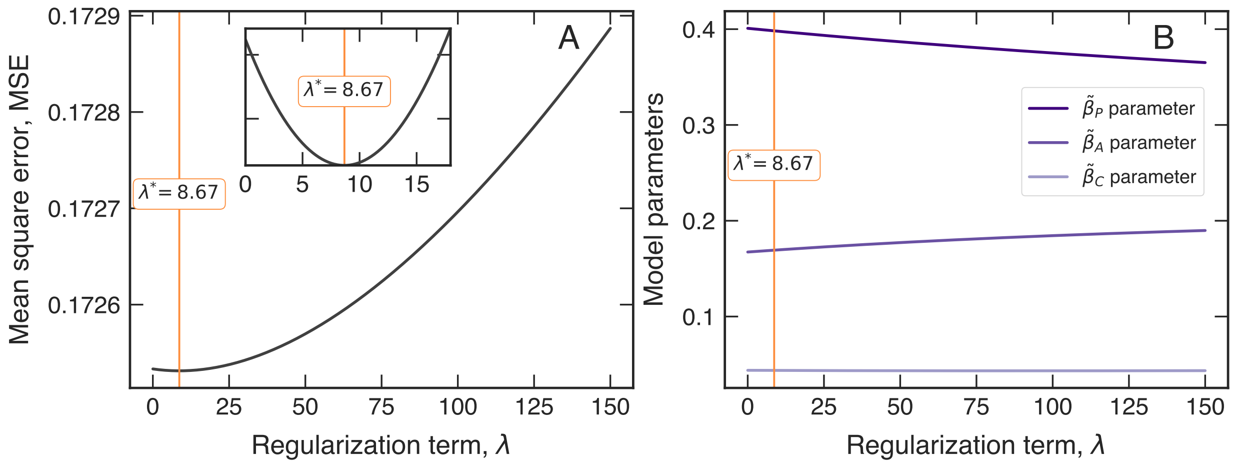

Supplementary Figure 18. **Fitting Eq. (5) [translog model] to data with the ridge regression approach.** (A) The black curve shows the dependence of the mean square error (MSE) on the regularization term (λ). The average value is estimated using a leave-one-out cross validation strategy. The vertical line indicates the minimum value of the MSE occurring at $\lambda = \lambda^* = 8.67$. The inset highlights the behavior around the minimum. (B) Dependence of the parameters $\tilde{\beta}_P$, $\tilde{\beta}_A$, and $\tilde{\beta}_C$ on the regularization term (λ). The optimal values for $\lambda = \lambda^*$ are $\tilde{\beta}_P = 0.40 \pm 0.02$, $\tilde{\beta}_A = 0.17 \pm 0.02$, and $\tilde{\beta}_C = 0.044 \pm 0.006$.

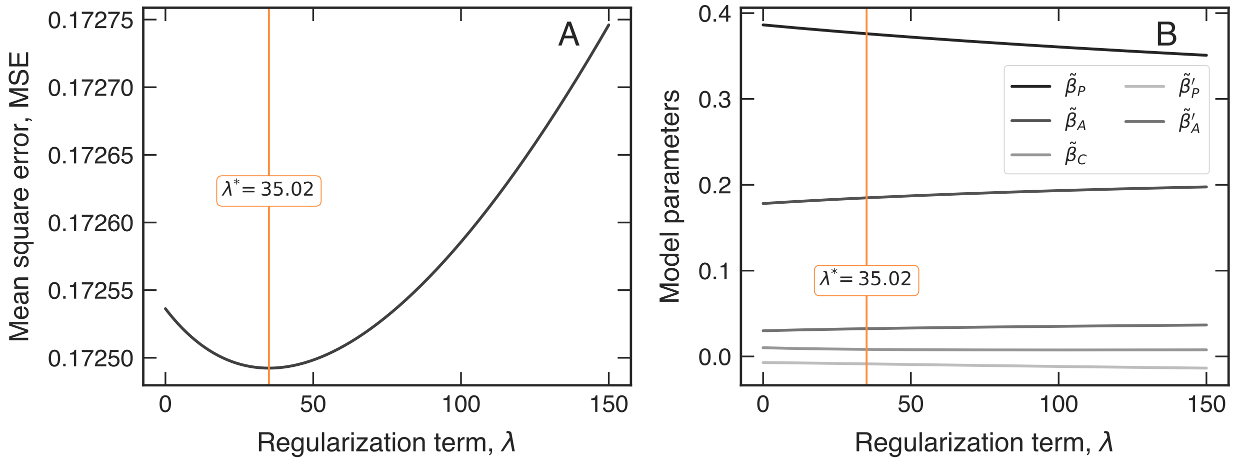

Supplementary Figure 19. **Fitting Eq. (20) [full translog model] to data with the ridge regression approach.** (A) The black curve shows the dependence of the mean square error (MSE) on the regularization term (λ). The average value is estimated using a leave-one-out cross validation strategy. The vertical line indicates the minimum value of the MSE occurring at $\lambda = \lambda^* = 35.02$. (B) Dependence of the parameters $\tilde{\beta}_P$, $\tilde{\beta}_A$, $\tilde{\beta}_C$, $\tilde{\beta}'_P$, and $\tilde{\beta}'_A$ on the regularization term (λ). The optimal values for $\lambda = \lambda^*$ are $\tilde{\beta}_P = 0.378 \pm 0.015$, $\tilde{\beta}_A = 0.184 \pm 0.020$, $\tilde{\beta}_C = 0.009 \pm 0.039$, $\tilde{\beta}'_P = -0.009 \pm 0.021$, and $\tilde{\beta}'_A = -0.032 \pm 0.020$.

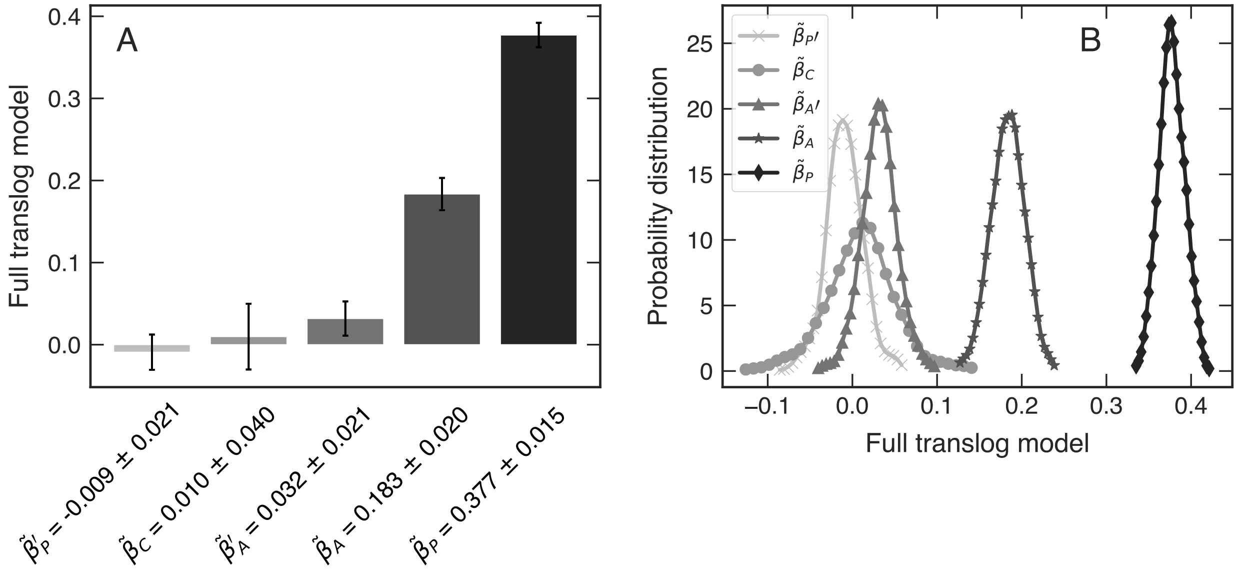

Supplementary Figure 20. **Estimating the errors in the parameters of Eq. (20) [full translog model].** (A) The bar plot shows the average values of the parameters $\tilde{\beta}_P$, $\tilde{\beta}_A$, $\tilde{\beta}_C$, $\tilde{\beta}'_P$, and $\tilde{\beta}'_A$ estimated after fitting the model to 1000 random samples (with replacement) of our data. Error bars stand for the standard deviation of these values. (B) Probability distribution of the values of $\tilde{\beta}_P$, $\tilde{\beta}_A$, $\tilde{\beta}_C$, $\tilde{\beta}'_P$, and $\tilde{\beta}'_A$ over all random samples. The permutation test on the model coefficients rejects the null hypothesis that $\tilde{\beta}_P$, $\tilde{\beta}_A$ and $\tilde{\beta}'_A$ are equal to zero; but cannot reject the same hypothesis for $\tilde{\beta}'_P$ and $\tilde{\beta}_C$ at the 95% confidence level.

Method, reference	Country	Year	Urban Units	Sample Size	Sector	Value (Lower,Upper)
						β
RMA[20]	Annex I	2005	Large Cities	22	N/A	0.87 (0.59, 0.95)
RMA[20]	Non-Annex I	2005	Large Cities	39	N/A	1.18 (0.90, 1.49)
OLS[19]	US	1999-2011	Metropolitan Areas	1875	Local CO2 and five air pollutants	0.75 (0.71, 0.79)
OLS[16]	US	1982-2011	Urban Centers	101	Excess CO_2 from congestion	1.27 (1.21, 1.33)
PW[16]	US	1982-2011	Urban Centers	101	Excess CO_2 from congestion	1.14 (1.38, 1.44)
OLS[17]	US	N/A	N/A	122	N/A	0.92 (0.90, 0.95)
OLS[17]	India	N/A	N/A	42	N/A	1.13 (1.02, 1.23)
OLS[17]	UK	N/A	N/A	35	N/A	0.76 (0.68, 0.83)
OLS[13]	GB	2010	City & Administrative Boundaries	28	Transport CO_2 emissions	1.02 (0.96, 1.08)
OLS[11]	US	2010	Metropolitan Areas	101	Excess CO_2 from congestion	1.26 (1.17, 1.35)
OLS[11]	OECD Countries	2014	Metropolitan Areas	268	Transport CO_2 emissions	1.21 (1.11, 1.31)
OLS[9]	US	2002	CCA clusters (l = 5 km, D = 1000)	2281	All Vulcan sectors	1.38 (1.35, 1.41)
OLS[9]	US	2002	CCA clusters (l > 10 km, 1000 < D < 4000)	N/A	All Vulcan sectors	1.46 (1.44, 1.48)
OLS[8]	US	1999-2008	Metropolitan Areas	933	All Vulcan sectors	0.93 (0.92, 0.95)
						α
OLS[15]	US	2000	CCA/GRUMP/GLC (l = 1 km)	4585	Buildings and on-road emissions	−0.78 (N/A, N/A)
OLS[15]	US	2000	CCA/GRUMP/GLC (l = 5 km)	3285	Buildings and on-road emissions	−0.79 (N/A, N/A)
OLS[15]	US	2000	CCA/GRUMP/GLC (l = 10 km)	2786	Buildings and on-road emissions	−0.82 (N/A, N/A)
OLS[15]	US	2000	CCA/GRUMP (l = 1 km)	5182	Buildings and on-road emissions	−0.90 (N/A, N/A)
OLS[15]	US	2000	CCA/GRUMP (l = 5 km)	2156	Buildings and on-road emissions	−1.10 (N/A, N/A)
OLS[15]	US	2000	CCA/GRUMP (l = 10 km)	1538	Buildings and on-road emissions	−1.13 (N/A, N/A)
OLS[5]	Global	1980	World cities	32	Gasoline consumption	−0.92 (−0.85, −0.99)
OLS[23]	EU	2007-2009	European cities	134	Transport CO_2 emissions	−0.19 (N/A, N/A)
OLS[23]	EU	2007-2009	France	24	Transport CO_2 emissions	−0.39 (N/A, N/A)
OLS[23]	EU	2007-2009	Spain	22	Transport CO_2 emissions	−0.40 (N/A, N/A)

Supplementary Table 1. Exponents associated with the urban scaling (β) and per capita density scaling (α). Abbreviations: Reduced Major Axis Regression (RMA), Ordinary Least Square Regression (OLS), Prais-Winsten Regression (PW).

**Property**	**Cobb-Douglas model, Eq. (3)**	**Translog model, Eq. (5)**
Elasticity of scale in terms of P and A	$\beta_P + \beta_A$	$\beta_P + \beta_A + \beta_C \log(PA)$
Elasticity of scale in terms of P and P/A	β_P	$\beta_P + \beta_C \log A$
Technical rate of substitution between P and A	$-\frac{\beta_P}{\beta_A(P/A)}$	$\frac{-1}{(P/A)}\left(\frac{\beta_P+\beta_C \log A}{\beta_A+\beta_C \log P}\right)$
Elasticity of substitution	1	$-\frac{\beta_P+\beta_A+\beta_C \log(PA)}{C(\beta_P+\beta_A+\beta_C \log(PA)-2\beta_C)}$
Marginal product of population (in terms of P and A)	β_P	$\beta_P + \beta_C \log A$
Marginal product of area (in terms of P and A)	β_A	$\beta_A + \beta_C \log P$
Marginal product of population (in terms of P and P/A)	$\beta_P + \beta_A$	$\beta_P + \beta_A + \beta_C \log(PA)$
Marginal product of density (in terms of P and P/A)	$-\beta_A$	$-\beta_A - \beta_C \log P$
Condition for population dominates over density	$\lvert\beta_P + \beta_A\rvert > \lvert\beta_A\rvert$	$A > 10^{-\beta_P/\beta_A}$ (if $\beta_P, \beta_A, \beta_C > 0$ and $P, A > 1$)
Condition for decreasing returns to scale (in terms of P and A)	$\beta_P + \beta_A < 0$	$PA < 10^{\frac{1-\beta_P-\beta_A}{\beta_C}}$
Condition for decreasing returns to scale (in terms of P and P/A)	$\beta_P < 0$	$A < 10^{\frac{1-\beta_P}{\beta_P}}$

Supplementary Table 2. Summary of the main properties of Cobb-Douglas [Eq. (3)] and translog [Eq. (5)] models. All marginal products are expressed in logarithmic scale.
